# Prognostic Factors for COVID-19 Hospitalized Patients with Preexisting Type 2 Diabetes

**DOI:** 10.1155/2022/9322332

**Published:** 2022-01-17

**Authors:** Yuanyuan Fu, Ling Hu, Hong-Wei Ren, Yi Zuo, Shaoqiu Chen, Qiu-Shi Zhang, Chen Shao, Yao Ma, Lin Wu, Jun-Jie Hao, Chuan-Zhen Wang, Zhanwei Wang, Richard Yanagihara, Youping Deng

**Affiliations:** ^1^Department of Quantitative Health Sciences, John A. Burns School of Medicine, University of Hawaii at Manoa, Honolulu, HI, USA; ^2^Tianyou Hospital, Affiliated to Wuhan University of Science and Technology, Wuhan, Hubei, China; ^3^Cancer Epidemiology Program, University of Hawaii Cancer Center, University of Hawaii at Manoa, Honolulu, HI, USA; ^4^Department of Pediatrics, John A. Burns School of Medicine, University of Hawaii at Manoa, Honolulu, HI, USA

## Abstract

**Background:**

Type 2 diabetes (T2D) as a worldwide chronic disease combined with the COVID-19 pandemic prompts the need for improving the management of hospitalized COVID-19 patients with preexisting T2D to reduce complications and the risk of death. This study aimed to identify clinical factors associated with COVID-19 outcomes specifically targeted at T2D patients and build an individualized risk prediction nomogram for risk stratification and early clinical intervention to reduce mortality.

**Methods:**

In this retrospective study, the clinical characteristics of 382 confirmed COVID-19 patients, consisting of 108 with and 274 without preexisting T2D, from January 8 to March 7, 2020, in Tianyou Hospital in Wuhan, China, were collected and analyzed. Univariate and multivariate Cox regression models were performed to identify specific clinical factors associated with mortality of COVID-19 patients with T2D. An individualized risk prediction nomogram was developed and evaluated by discrimination and calibration.

**Results:**

Nearly 15% (16/108) of hospitalized COVID-19 patients with T2D died. Twelve risk factors predictive of mortality were identified. Older age (HR = 1.076, 95% CI = 1.014–1.143, *p*=0.016), elevated glucose level (HR = 1.153, 95% CI = 1.038–1.28, *p*=0.0079), increased serum amyloid A (SAA) (HR = 1.007, 95% CI = 1.001–1.014, *p*=0.022), diabetes treatment with only oral diabetes medication (HR = 0.152, 95%CI = 0.032–0.73, *p*=0.0036), and oral medication plus insulin (HR = 0.095, 95%CI = 0.019–0.462, *p*=0.019) were independent prognostic factors. A nomogram based on these prognostic factors was built for early prediction of 7-day, 14-day, and 21-day survival of diabetes patients. High concordance index (C-index) was achieved, and the calibration curves showed the model had good prediction ability within three weeks of COVID-19 onset.

**Conclusions:**

By incorporating specific prognostic factors, this study provided a user-friendly graphical risk prediction tool for clinicians to quickly identify high-risk T2D patients hospitalized for COVID-19.

## 1. Introduction

Coronavirus disease 2019 (COVID-19), which is caused by a novel betacoronavirus (named severe acute respiratory syndrome, coronavirus 2, or SARS-CoV-2), has been declared a global pandemic by the World Health Organization [1, 2]. Studies report that 20–50% of COVID-19 patients have diabetes depending on different areas [3], and COVID-19 patients with diabetes have worse outcomes than nondiabetic patients [4–7]. Diabetes as a chronic disease affects more than 463 million people worldwide [8, 9]. Its high prevalence combined with the poor COVID-19 clinical outcomes prompts the need for improving the management of diabetic patients to reduce complications and the risk of death.

Type 2 diabetes (T2D) accounts for 90–95% of all diabetes globally [10]. T2D patients may deteriorate more rapidly once infected due to the weakened immune system [1, 11]. The COVID-19 pandemic represents a serious threat to this large vulnerable population. In this study, we identified clinical factors associated with COVID-19 outcomes specifically targeted at T2D patients and built a nomogram for clinicians to quickly identify high-risk T2D patients hospitalized for COVID-19 and for patients to easily do self-monitoring through risk assessment.

## 2. Research Design and Methods

### 2.1. Study Design and Participants

A retrospective cohort study was conducted of sequentially hospitalized COVID-19 patients from January 8 to March 7, 2020 in Tianyou Hospital, affiliated to the Wuhan University of Science and Technology in China. The final follow-up date was March 18, 2020. Patients were included based on the following criteria: (1) age ≥18 years, (2) chest computer tomography (CT) scans were performed at the time of admission and discharge, (3) blood glucose and other routine laboratory tests were performed on admission, and (4) treatments, complications, and definitive clinical outcomes (discharged or died) were clearly recorded between admission and the final follow-up date. This study was approved by the institutional ethics board of Tianyou Hospital, affiliated to the Wuhan University of Science and Technology (Ethical application no.: WKDTY-2020039).

### 2.2. Date Collection

Demographics, symptoms, comorbidities, laboratory tests, treatments, disease progression, and outcomes of COVID-19 patients with and without T2D were collected from electronic medical records (EMR). Information about age of diabetes diagnosis, duration of diabetes, and diabetes treatment was collected from diabetic patients. All raw data were independently verified by two experienced physician teams to guarantee accuracy. The endpoint was in-hospital death of COVID-19 patients.

### 2.3. Case Definition

All patients were diagnosed with COVID-19 according to the WHO interim guidance [12]. Patients with SARS-CoV-2 infection can experience a range of clinical manifestations, and a patient's clinical status may change over time. Based on the National Institutes of Health (US) Coronavirus Disease 2019 (COVID-19) Treatment Guidelines [13], the patients were grouped into the following severity of illness categories according to their clinical presentation on admission: nonsevere (patients who showed no symptoms or fever, respiratory symptoms, and CT evidence of pneumonia); severe (patients who showed features of nonsevere patients as well as respiratory distress with respiratory rate ≥30 breaths/min, oxygen saturation less than 94% on room air at sea level, arterial partial pressure of oxygen/oxygen concentration less than 300 mmHg, or lung infiltrates >50%); and critical (patients who showed respiratory failure requiring ventilatory support as well as shock and organ dysfunction requiring intensive care and/or multiple organ dysfunction). T2D status was designated based on the patient's medical history and defined according to the guidelines of Chinese Diabetes Society [14]. All T2D patients took diabetes medication regularly in the past year and did not show obvious symptoms of acute cardiac injury on admission. Acute cardiac injury was defined as electrocardiographic and echocardiographic abnormalities or by cardiac biomarker (hypersensitive troponin I or creatine kinase) elevation above 99% of the upper reference limit [15]. Disease favorable outcome was defined as full recovery and discharge, progression from critical/severe to nonsevere disease status, and/or maintenance of nonsevere status; conversely, unfavorable outcomes included death, progression from nonsevere to severe/critical or severe to critical, and/or maintenance of severe or critical status.

### 2.4. Statistical Analysis

Categorical variables were summarized as numbers with percentages, and continuous variables were presented as medians with interquartile range (IQR). Student's *t*-test or Wilcoxon rank-sum test was used for continuous variables. The chi-square test or Fisher exact test was used for categorical variables as appropriate. Hazard ratios (HRs) and 95% confidence intervals (CIs) were calculated by univariate and multivariate Cox proportional hazard regression models. A final model selection was performed via a backward stepwise selection process. Time-dependent receiver operating characteristic curve (ROC) and the calculated time-dependent area under the curve (AUC) were used to characterize the discrimination potential of the Cox regression model [16, 17]. A predictive nomogram was developed to generate a combined indicator for estimating the mortality risk, and it was validated by bootstrap resampling 1,000 times. Discrimination was used to evaluate the model's ability to separate patients with different outcomes and quantified using Harrell's concordance index (C-index) [18, 19]. Calibration was used to test how close the predictions were to the actual outcomes by demonstrating calibration curves.

If the number of events was too small to calculate the HR, the variables were excluded. Cumulative rates of in-hospital deaths were determined using the Kaplan–Meier method and Log-rank test. The survival time was defined as the interval from the date of admission to the date of death. The analyses were performed by using R software (version 3.6.1, R Foundation, Vienna, Austria) or Statistical Analysis System (SAS) software (version 9.4, SAS Institute Inc., Cary, NC), with a statistical significance set at two-sided *p* < 0.05.

## 3. Results

### 3.1. Participants and Clinical Outcomes

**Figure 1:**
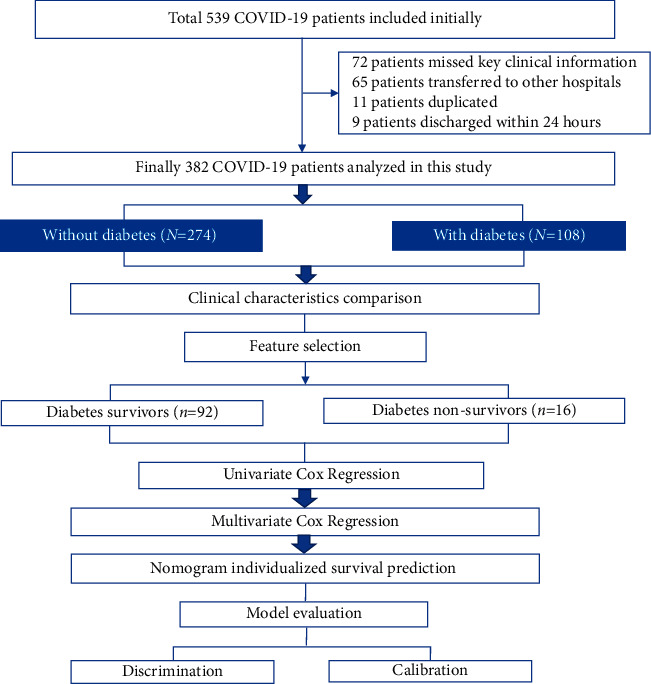
Study design. A total of 382 hospitalized COVID-19 patients (108 with and 274 without T2D) were included in this study. In comparing the clinical factors between COVID-19 patients in the diabetes and nondiabetes groups and between survivors and nonsurvivors, clinical factors specifically associated with disease outcomes were used to perform univariate and multivariate Cox regression models. Based on the independent factors identified by Cox models, a nomogram was built for individualized survival prediction and validated by discrimination and calibration.

The initial 539 confirmed COVID-19 patients were identified from January 8 to March 7, 2020 in Tianyou Hospital in Wuhan, China. A total of 157 patients were excluded due to incomplete or duplicate medical records (72 patients missed key clinical information, 11 patients were duplicated, 65 patients were transferred to other hospitals, and 9 patients were discharged within 24 hours), leaving 382 patients with a definitive clinical outcome (discharged or died) in this study, and 108 of them had T2D. The median age was 63 (range: 23–91) years; 49.5% (189/382) were female and 50.5% (193/382) were male. About 9.7% (37/382) of patients died during hospitalization. There were 8.6% (33/382) critical patients, 49.5% (189/382) severe patients, and 41.9% (160/382) nonsevere patients on admission. The median follow-up time was 20 days. The final follow-up date was March 18, 2020. The average follow-up time was 19 days. Of the 108 COVID-19 patients with T2D, 92 (85.2%) patients recovered and were discharged, and 16 (14.8%) patients died during the study period. The study design is shown in Figure 1.

### 3.2. Comparison of Clinical Features between COVID-19 Patients with and without T2D

The death rate among the 108 patients with T2D (14.8%) was two times higher than that among the 274 patients without T2D (7.7%). In Table S1, the complete clinical and biochemical panel provides the comparison of both COVID-19 patients with and without T2D, delineating the possible indicators predisposing patients with T2D to have worse clinical outcomes. The significant comparison results of COVID-19 patients with and without T2D were summarized in Table 1.

The median ages of the diabetes and nondiabetes groups were 68 and 60 years, respectively. Moreover, 41.7% of diabetic patients were unemployed and only 12.0% were employed. Hypnotics were more commonly administered to nondiabetic patients (28.6%) than to diabetic patients (16.2%). Fever and cough were the most common symptoms, with the former being more common in the nondiabetes group (85.4% vs. 66.7%) and the latter more common in the diabetes group (62.0% vs. 49.3%). The diabetes group had a significantly higher rate of preexisting comorbidities (63.9%, *p* < 0.0001), with hypertension in 60.2% and cardiovascular disease in 19.4%. As for disease severity, more than half of the diabetes patients showed severe disease, and 17.6% were critically ill on admission, compared to nondiabetes patients (5.1%).

COVID-19 patients with T2D were more likely to show no ground-glass opacity (GGO) (13.9% vs. 2.6%, *p* < 0.0001) and diffuse patchy ground glass and air bronchogram (12.0% vs. 5.8%, *p*=0.039). By contrast, 53.7% of COVID-19 patients without T2D showed bilateral GGO. Significant differences were found in the white blood cell count (*p* = 0.011), neutrophil count (*p* < 0.0001), lymphocyte count (*p* < 0.0001), monocyte count (*p* < 0.0001), aspartate aminotransferase (AST) (*p*=0.029), serum amyloid A (SAA) (*p*=0.016), hypersensitive troponin I (*p*=0.011), creatinine (*p* = 0.0023), procalcitonin (*p* < 0.0001), blood glucose (*p* < 0.0001), potassium (*p*=0.0073), and low-density lipoprotein (LDL) cholesterol level (*p*=0.047).

Most patients received antiviral therapy, with ganciclovir (65.9%), arbidol (65.6%), and oseltamivir (64.0%) being the most frequently administered. Oseltamivir (*p* < 0.0001) and ganciclovir (*p*=0.0075) were more often used in the nondiabetes group. Antibiotics were administered to 94.9% of nondiabetes patients and 78.7% of diabetes patients (*p* < 0.0001). Regarding oxygen therapy, more COVID-19 patients with T2D used noninvasive ventilation and invasive ventilation. Acute respiratory distress syndrome (ARDS), acute cardiac injury, arrhythmia, and septic shock were the most common complications. COVID-19 patients with T2D had a greater occurrence of arrhythmia (13.9% vs. 4.8%) and septic shock (10.2% vs. 2.9%).

### 3.3. Comparison of Clinical Features between Survivors and Nonsurvivors with T2D

The results were shown in Table 1 (a complete version is available as Supplementary table S2). Referring to the classification based on patients' severity of the SARS-CoV-2 infection at hospital admission in the “case definition” method, 108 COVID-19 patients with T2D (55 females and 53 males) were characterized into nonsevere (32, 29.6%), severe (57, 52.8%), and critical (19, 17.6%) groups. Patients in the critical group made up the majority (56.2%) of the nonsurvivors. The median age of patients in the survivor group was 67 years (IQR, 60–73), which was significantly lower than that in the nonsurvivor group (74 years) (IQR, 65–89, *p*=0.017). The patients' age at diagnosis with diabetes was not significantly different between the survivors and nonsurvivors. For their duration of diabetes, the median was 8 years (IQR, 4–13). There were more obese patients (BMI≥30) in the nonsurvivor group than the survivor group (14.3% vs. 1.3%). On admission, fever (72, 66.7%) and cough (67, 62.0%) were the most common symptoms, while dyspnea (5, 4.6%) and nausea and vomiting (4, 3.7%) were uncommon. The majority of COVID-19 patients with T2D had at least one coexisting illness (69/108, 63.9%), as did 87.5% (14/16) of the nonsurvivors.

Chest CT abnormalities were found in all 108 COVID-19 patients with T2D, with bilateral GGO in 40.7% (44/108), while no significant differences were found between the survivors and nonsurvivors. Representative CTs are shown in Figure S1. Laboratory findings between nonsurvivors and survivors showed significant differences in the white blood cell count (*p*=0.011), neutrophil count (*p*=0.0029), platelet count (*p*=0.036), AST (*p* < 0.0001), CRP (*p* < 0.0001), SAA (*p* < 0.0001), activated partial thromboplastic time (*p*=0.0044), d-dimer (*p*=0.011), lactate dehydrogenase (*p*=0.0011), creatinine (*p*=0.026), blood glucose (*p*=0.05), LDL (*p*=0.012), and HDL (*p*=0.013) (Table S2).

During hospitalization, all T2D patients received diabetes treatment, either only oral medication, oral medication plus insulin, or only insulin. The clinical outcomes were significantly different between the three diabetes treatment groups (*p* < 0.0001). In the nonsurvivor group, most (75.0%) were treated with only insulin, and only two patients died with oral medication only and oral medication plus insulin treatment. The majority of the survivor group (68.5%) were treated by oral medication alone, with 17.4% of them using insulin only.

Arbidol (64.8%), ganciclovir (55.6%), and oseltamivir (47.2%) were the three most frequently used antiviral medications. Most of antivirus therapy with COVID-19 did not significantly improve patient outcomes except for oseltamivir (*p*=0.028) and ganciclovir (*p*=0.0059). Antibiotics were administered to 78.7% of patients. Patients given corticosteroids and glucocorticoids had poor clinical outcomes (*p* < 0.0001).

In the nonsurvivor group of COVID-19 patients with T2D, ARDS (93.8% vs. 16.3%, *p* < 0.0001), acute cardiac injury (62.5% vs. 4.4%, *p* < 0.0001), arrhythmia (50% vs. 7.6%, *p*=0.0001), and septic shock (50% vs. 3.3%, *p* < 0.0001) were significantly more common than in the survivor group.

### 3.4. Prognostic Factors Specifically for COVID-19 Patients with T2D

**Figure 2:**
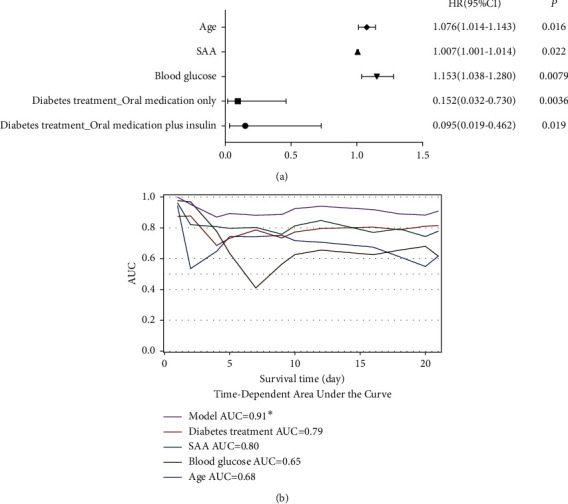
Multivariate Cox regression model and time-dependent ROC. (a) Factors showing significant independent association with mortality. The hazard ratio, 95% CI, and *p* values are derived from multivariate Cox proportion hazard regression modelling. (b) Time-dependent area under the curve (AUC), demonstrating the model has a good discriminatory ability of 91%.

After comparing the clinical factors between patients with and without T2D as well as survivors and nonsurvivors in the T2D cohort. The factors specifically associated with T2D patients' outcomes were input to perform the univariate Cox model, the following 12 variables of age, duration of diabetes, disease severity, cirrhosis, cardiovascular and cerebrovascular diseases, neutrophil count, AST, SAA, blood glucose, diabetes treatment, ganciclovir, and steroid therapy were identified as prognostic factors for the COVID-19 patients with T2D (Table 2). Furthermore, multivariate Cox regression analysis indicated that diabetes treatment of oral medication only (HR = 0.152, 95%CI = 0.032–0.73, *p*=0.0036) or oral medication plus insulin (HR = 0.095, 95%CI = 0.019–0.462, *p*=0.019)), older age (HR = 1.076, 95% CI = 1.014–1.143, *p*=0.016), elevated glucose level (HR = 1.153, 95% CI = 1.038–1.28, *p*=0.0079), and increased SAA (HR = 1.007, 95% CI = 1.001–1.014, *p*=0.022) are independent prognostic factors (Figure 2(a)). Time-dependent ROC showed the AUC of the model was 91%, indicating the excellent discriminatory ability of the model (Figure 2(b)).

### 3.5. Development and Validation of Individualized Prediction Nomogram

**Figure 3:**
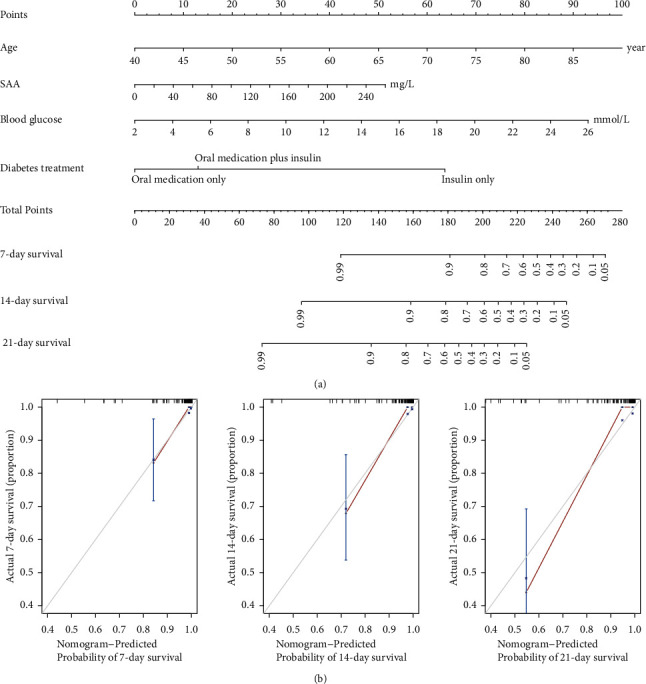
Development and performance of a nomogram to predict the survival probability of COVID-19 patients with T2D. (a) Prognostic nomogram for predicting the overall survival probability of COVID-19 patients with T2D. To use the nomogram, an individual patient's value is located on each variable axis, and a line is drawn upward to determine the number of points received for each variable. The sum of these numbers is located on the total point axis, from where a line can be drawn downward to predict the survival probability at 7, 14, and 21 days of hospitalization. (b) To evaluate the nomogram model, the calibration curves are shown predicting the likelihood of 7-day, 14-day, and 21-day survival. The *x*-axis displays the predicted probabilities generated by the statistical model, and the *y*-axis shows the fraction of patients who were alive at the given predicted probability. The diagonal grey line represents a perfect prediction by an ideal model. The solid red line represents the performance of the nomogram, of which a closer fit to the grey line represents a better prediction. As demonstrated, there was an excellent correspondence between the predicted probability of 7-day survival and the observed frequency of survival in COVID-19 patients with T2D.

The nomogram was constructed to predict the mortality risk of COVID-19 patients with T2D according to the final multivariate analysis. Each independent factor was assigned a score based on the point scale at the top of the nomogram, and the total points were calculated. Patient 7-, 14-, and 21-day mortality probabilities were obtained from the bottom point scale of the nomogram (Figure 3(a)). The C-index of the nomogram was 0.90 (95% CI, 0.84–0.96) and a high C-index value of 0.87 was reached in the internal validation assessed by bootstrapping (1,000 replicates), suggesting that the prediction of the nomogram was consistent with the actual observation for COVID-19 patients with T2D. The calibration plots based on the bootstrap resampling method showed the model had a good agreement between predicted risk and actual proportions at 7, 14, and 21 days (Figure 3(b)).

### 3.6. Potential Benefits and Detriments of Diabetes Medication

**Figure 4:**
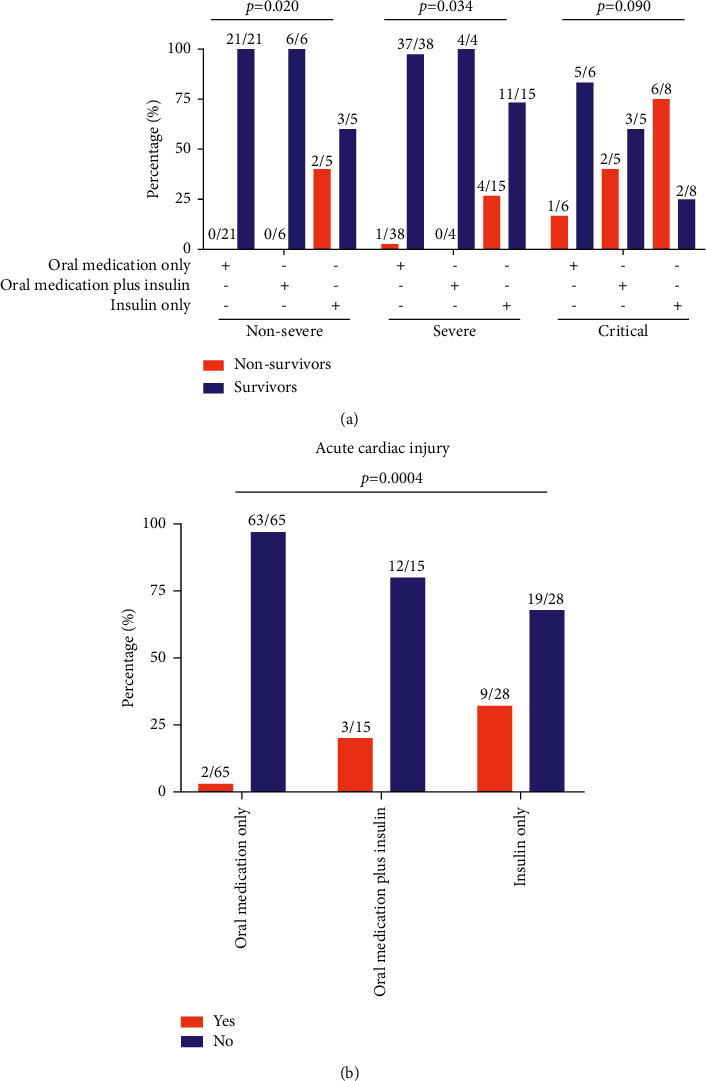
Clinical outcomes and complications of acute cardiac injury in three different diabetes treatment groups. (a) Clinical outcomes of patients in the nonsevere, severe, and critical disease groups. The percentages are calculated by dividing the number of nonsurvivors and survivors by the total number of patients in each disease severity group. (b) Complications of acute cardiac injury in different treatment groups of COVID-19 patients with T2D. The percentages are calculated by dividing the number of nonsurvivors and survivors by the total number of the complication status with either acute cardiac injury (yes) or not (no).

The diabetes treatments for COVID-19 patients with T2D during hospitalization were counted: 28 (25.9%) only insulin, 15 (13.9%) one or more oral medications, and 65 (60.2%) oral medications plus insulin. Based on the multivariate Cox model, COVID-19 patients with diabetes treated with oral medication only or with oral medication plus insulin had a significant lower mortality risk than those treated with insulin only. The survival curves for different diabetes treatments are shown in Figure S2. Considering the different disease severity of COVID-19 on admission may have a different reaction to diabetes treatment, COVID-19 patients with T2D were analyzed according to clinical disease severity to further validate the effect of diabetes medication on clinical outcomes. In the nonsevere and severe disease groups, favorable outcomes were more prevalent among COVID-19 patients receiving oral medication only or oral medication plus insulin than among the insulin-only group (100%, 100% vs. 60% for nonsevere, *p*=0.020; 97.4%, 100% vs. 73.3% for severe, *p*=0.034) (Figure 4(a)). In the critical disease group, favorable outcomes were still more prevalent among COVID-19 patients with oral medication only and oral medication plus insulin but was not statistically significant (83.3%, 60% vs. 25% for critical, *p*=0.090) (Figure 4(a)). We also compared the blood glucose level of different diabetes treatment groups. As show in Figure S3, the blood glucose level of patients treated with oral medication plus insulin was higher than that of patients treated with insulin only. The insulin-only group had significantly increased acute cardiac injury (*p*=0.0004) (Figure 4(b)).

**Figure 5:**
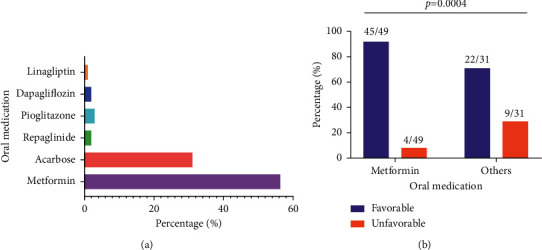
Oral medication and association with disease development. (a) A total of six types of oral medication was used in our cohort. (b) The proportion of metformin was significantly higher in the favorable outcome group than that of the other oral medications (91.8% vs. 71.0%, *p*=0.0004).

As shown in Figure 5(a), six types of diabetes oral medication were used in this cohort, including metformin (49, 52.7%), alpha-glucosidase inhibitors (acarbose) (31, 33.3%), meglitinides (repaglinide) (7, 7.5%), thiazolidinediones (pioglitazone) (3, 3.2%), sodium-glucose cotransporter 2 (SGLT2) inhibitors (dapagliflozin) (2, 2.2%), and dipeptidyl-peptidase 4 (DPP-4) inhibitors (linagliptin) (1, 1.1%). The use of metformin was significantly associated with favorable outcomes compared with other oral medications (91.8% vs. 71.0%, *p*=0.0004) (Figure 5(b)).

## 4. Discussion

In this study, we identified prognostic factors in patients with T2D hospitalized for COVID-19 and developed a nomogram for predicting the individual mortality risk for this special patient group.

In our study, we found the death rate among the patients with T2D was twice as high as that among the patients without T2D which might be related to the higher prevalence of coexisting comorbidities and older age in T2D patients. These findings are consistent with previous reports that older age and comorbidities are contributors to increased risk of COVID-19 susceptibility, disease severity, and clinical outcomes [4, 20–24]. Comorbidities could be considered a consequence of diabetes and have a synergistic effect with age on the mortality risk of patients with T2D.

The blood glucose level has been reported to be an important prognostic factor for COVID-19 progression and fatality from a large retroactive study in China [25]. High glucose level contributes to acute respiratory distress syndrome risk in COVID-19 patients and other comorbidities. One study showed 1 mmol/L increase in plasma glucose level could increase 6% risk of hospitalization for pneumonia [26]. The elevated blood glucose could also facilitate the entire process of the COVID-19 infection [27]. COVID-19 patients with T2D have a higher blood glucose level than those without T2D leading to the severe adverse clinical outcome.

As identified in our model, oral diabetes medication may offer a protective impact on COVID-19. This may be achieved through the medication's action on the receptor of SARS-CoV-2. It is worthwhile to explore the interactions of SARS-CoV-2 with commonly used diabetes medications, with the potential to repurpose them to prevent COVID-19 disease progression. Angiotensin-converting enzyme 2 (ACE2) is the principal cellular receptor for the novel SARS-CoV-2, mainly in cell membranes of lung alveolar epithelial cells and enterocytes of the small intestine, which bind with the receptor-binding domain (RBD) of the spike protein (S) on the virus surface [28–31]. In diabetes, ACE2 has been suggested as a potential therapeutic target for the management of diabetes and its complications [32, 33] and reduction of the risk of type 2 diabetes development [34]. Metformin may have protective effects against SARS-CoV-2 by working with ACE2 in two ways [35, 36]. First, metformin can activate the AMP-activated protein kinase (AMPK) signaling pathway, leading to phosphorylation of ACE2. Theoretically, conformational and structural changes in ACE2 may lead to decreased binding to SARS-CoV-2 RBD due to steric hindrance by the addition of a phosphate group [37]. Second, AMPK phosphorylation of ACE2 enhances ACE2 stability, which may prevent SARS-CoV-2-mediated ACE2 downregulation [38, 39]. Thus, metformin may decrease the ACE2 binding ability of SARS-CoV-2 as well as increase the expression and stability of ACE2 through activating AMPK. Metformin can also be involved in the development of COVID-19 by inhibiting the mTOR signaling pathway [40, 41] and altering the composition of the gut microbiota [42]. A recent study showed users of metformin had few adverse outcomes compared to nonusers [43]. Continued use of metformin reduced the risk of death or ICU admission during hospitalization [44].

Previous studies indicated that insulin therapy may be associated with increased cardiovascular events and mortality [45, 46].Treatment with regular insulin may result in postmeal hyperglycemia and an increased risk of late postprandial hypoglycemia. Hypoglycemia is associated with an increased risk of cardiac arrhythmia due to a mismatch between insulin and carbohydrate intake, alcohol, or exercise [47]. Additionally, hypoglycemia can stimulate the catecholamine release and prolong the QT interval, which can lead to adverse cardiovascular events [48]. The use of insulin, therefore, should be closely monitored in COVID-19 patients with T2D.

SARS-CoV-2 infection can deregulate the immune response, which may trigger viral hyperinflammation in patients with severe COVID-19 [49] and increase the risk for SAA amyloid formation and subsequent pathologies [50]. SAA is a nonspecific acute phase protein mainly produced by the cytokines IL-1*β*, IL-6, and TNF-*α* in liver cells, which increase in chronic inflammatory processes, as in diabetes and obesity. Previous studies showed that patients with severe acute respiratory syndrome had significantly increased levels of SAA, suggesting SAA could be used as a biomarker to monitor the progression of respiratory diseases [51]. Recent studies indicated that COVID-19 patients with significantly increased SAA levels had poor prognosis [7, 52], and SAA could be an excellent biomarker in discrimination between moderate and severe COVID-19 infection [53]. Meta-analysis also showed the severe COVID-19 patients had markedly higher SAA levels [54, 55]. High mortality risk of COVID-19 patients with diabetes may be due to the acute increase of SAA after virus infection.

In summary, our study provided a user-friendly graphical nomogram to help risk assessment for COVID-19 patients with T2D. By incorporating the prognostic factors of age, SAA, blood glucose, and diabetes treatment, this scoring tool could predict the probability of death at 7, 14, and 21 days. The potential protective effects of oral diabetes medication in COVID-19 highlighted a potential focus of future research.

## Figures and Tables

**Table 1:** Characteristics, radiologic and laboratory findings, treatments, and complications of COVID-19 patients with and without T2D, between survivors and nonsurvivors in COVID-19 patients with T2D.

Variables	All COVID-19 patients (*N* = 382)				COVID-19 patients with T2D (*n* = 108)		
	Total (*N* = 382)	Nondiabetes (*n* = 274)	Diabetes (*n* = 108)	*p* value	Nonsurvivor (*n* = 16)	Survivor (*n* = 92)	*p* value
Age, y	63 (52–70)	60 (48–67)	68 (61–75)	<0.0001	74 (65–89)	67 (60–73)	0.017
Age at diagnosed with diabetes, y	—	—	—	—	62 (56–67)	60 (51–66)	0.14
Duration of diabetes, y	—	—	—	—	10 (6–20)	8 (4–12)	0.051
*Occupation*							
Employee	116 (30.37)	103 (37.59)	13 (12.04)	<0.0001	1 (6.3)	12 (13.0)	0.17
Retired	161 (42.15)	111 (40.51)	50 (46.30)		11 (68.7)	39 (42.4)	
Unemployment	105 (27.49)	60 (21.90)	45 (41.66)		4 (25.0)	41 (44.6)	

*Signs and symptoms*							
Fever	306 (80.10)	234 (85.40)	72 (66.67)	<0.0001	9/16 (56.3)	63/92 (68.5)	0.34
Cough	202 (52.88)	135 (49.27)	67 (62.04)	0.024	9/16 (56.3)	58/92 (63.0)	0.59
Fever and cough	265 (69.37)	207 (75.55)	58 (53.70)	<0.0001	7/16 (43.7)	51/92 (55.4)	0.43
Chest distress	8 (2.09)	2 (0.73)	6 (5.56)	0.0076	2/16 (12.5)	4/92 (4.4)	0.21
Nausea and vomiting	4 (1.05)	0	4 (1.05)	0.0061	1/16 (6.3)	3/92 (85.2)	0.48

*Coexisting conditions*							
Any comorbidity	138 (36.31)	69 (25.18)	69 (63.89)	<0.0001	14/16 (87.5)	55/92 (59.8)	0.033
Hypertension	144 (37.70)	79 (28.83)	65 (60.19)	<0.0001	10/16 (62.5)	55/92 (59.8)	1
Cerebrovascular disease	14 (3.66)	4 (1.46)	10 (9.26)	0.0008	0/16 (0)	10/92 (10.9)	0.35
Cardiovascular and cerebrovascular diseases	48 (12.57)	27 (9.85)	21 (19.44)	0.011	8/16 (50.0)	13/92 (14.1)	0.0027
Endocrine system disease	30 (7.85)	7 (2.55)	23 (21.30)	<0.0001	1/16 (6.3)	22/92 (23.9)	0.18

*Disease severity*							
Nonsevere	160 (41.88)	128 (46.72)	32 (29.63)	<0.0001	2 (12.5)	30 (32.6)	0.0003
Severe	189 (49.48)	132 (48.18)	57 (52.78)		5 (31.3)	52 (56.5)	
Critical	33 (8.64)	14 (5.11)	19 (17.59)		9 (56.2)	10 (10.9)	

*Abnormalities on chest CT*							
No GGO	22 (5.76)	7 (2.55)	15 (13.89)	<0.0001	1/16 (6.3)	14/92 (15.2)	0.46
Bilateral GGO	191 (50.00)	147 (53.65)	44 (40.74)	0.023	6/16 (37.5)	38/92 (41.3)	1
Diffuse patchy ground glass and air bronchogram	29 (7.59)	16 (5.84)	13 (12.04)	0.039	1/16 (6.3)	12/92 (13.0)	0.69

*White blood cell count, 10* ^*9*^ */L*							
<4	98 (26.92)	74 (28.79)	24 (22.43)	0.011	3 (20.0)	21 (22.8)	0.011
4–10	240 (65.93)	164 (63.81)	76 (71.03)		8 (53.3)	68 (73.9)	
>10	26 (7.14)	19 (7.39)	7 (6.54)		4 (26.7)	3 (3.3)	

*Neutrophil count, 10* ^*9*^ */L*							
<40	127 (34.89)	60 (23.35)	67 (62.62)	<0.0001	5 (33.3)	62 (67.4)	0.0029
40–75	137 (37.64)	118 (45.91)	19 (17.76)		2 (13.3)	17 (18.5)	
>75	100 (27.47)	79 (30.74)	21 (19.63)		8 (53.4)	13 (14.1)	

*Lymphocyte count, 10* ^*9*^ */L*							
<20	238 (65.38)	147 (57.20)	91 (85.05)	<0.0001	14 (93.3)	77 (83.7)	0.46
20–50	122 (33.52)	106 (41.25)	16 (14.95)		1 (6.7)	15 (16.3)	
>50	4 (1.10)	4 (1.56)	0				

*Monocyte count, 10* ^*9*^ */L*							
<3	160 (44.20)	86 (33.73)	74 (69.16)	<0.0001	9 (60.0)	65 (70.7)	0.39
3–10	160 (44.20)	134 (52.55)	26 (24.30)		4 (26.7)	22 (23.9)	
>10	42 (11.60)	35 (13.73)	7 (6.54)		2 (13.3)	5 (5.4)	

*Aspartate aminotransferase, U/L*							
<13	50(13.40)	41 (15.30)	9 (8.57)	0.029	0	9 (10.0)	<0.0001
13-35	227(60.86)	152 (56.72)	75 (71.43)		5 (33.3)	70 (77.8)	
>35	96 (25.74)	75 (27.99)	21 (20.00)		10 (66.7)	11 (12.2)	

Serum amyloid A (SAA), mg/L	92.5 (22.0–210.0)	115 (34–210)	34 (4–200)	0.0016	210 (200–250)	153 (2–153.5)	**<**0.0001
Hypersensitive troponin I, pg/mL	0.008 (0–0.041)	0.008 (0–0.059)	0.007 (0.001–0.0225)	0.011	0.039 (0.01–0.13)	0.001 (0–0.017)	0.18
Creatinine, *μ*mol/L	63 (48–79)	60 (46–76)	68.5 (52.0–87.0)	0.0023	74 (66–116)	68 (49–86)	0.026
Procalcitonin, ng/mL	0.25 (0.01–0.06)	0.03 (0.01–0.07)	0.02 (0.01–0.06)	<0.0001	0.05 (0.02–0.23)	0.02 (0.01–0.05)	0.35
Blood glucose, mmol/L	5.8 (5.1–7.5)	5.5 (4.9–6.4)	7.7 (5.9–10.8)	<0.0001	11.5 (5.4–18.3)	7.6 (6.1–9.5)	0.05
Glycated hemoglobin, mmol/mol					7.7 (6.6–8.6)	7.5 (6.5–8.6)	0.73
Potassium, mmol/L	4.0 (3.6–4.4)	3.9 (3.5–4.3)	4.1 (3.7–4.5)	0.0073	4.2 (3.4–4.6)	4.1 (3.7–4.5)	0.47
LDL, mmol/L	2.31 (1.88–2.96)	2.26 (1.82–2.86)	2.50 (2.12–3.11)	0.047	2.3 (2.1–2.8)	2.6 (2.1–3.2)	0.012
*Diabetes treatment*							
Oral medication plus insulin	—	—	—	—	2 (12.5)	13 (14.1)	<0.0001
Oral medication only	—	—	—	—	2 (12.5)	63 (68.5)	
Insulin only	—	—	—	—	12 (75.0)	16 (17.4)	

*Antiviral therapy*							
Oseltamivir	244 (64.04)	193 (70.70)	51 (47.22)	<0.0001	12/16 (75.0)	39/92 (85.2)	0.028
Ganciclovir	251 (65.88)	191 (69.96)	60 (55.56)	0.0075	14/16 (87.5)	46/92 (50.0)	0.0059

*Antibiotic therapy*							
Antibiotics	344 (90.29)	259 (94.87)	85 (78.70)	<0.0001	16/16 (100)	69/92 (75.0)	0.021

Steroid therapy							
Oxygen support							
*Non-invasive ventilation*							
No	330 (86.39)	251 (91.61)	79 (73.15)	<0.0001	4 (25.0)	75 (81.5)	<0.0001
Yes	52 (13.61)	23 (8.39)	29 (26.85)		12 (75.0)	17 (18.5)	

*Complications*							
Arrhythmia	28 (7.35)	13 (4.76)	15 (13.89)	0.0021	8/16 (50.0)	7/92 (7.6)	0.0001
Septic shock	19 (4.99)	8 (2.93)	11 (10.19)	0.0034	8/16 (50.0)	3/92 (3.3)	<0.0001

**Table 2:** Univariate Cox proportional hazard regression analysis results

Variables	HR	95% CI		*P* value
Age, y	1.06	1.01	1.19	**0.025**
Duration of diabetes, y	1.08	1.01	1.14	**0.017**
*Disease severity*				
Nonsevere	1			
Severe	1.16	0.22	5.98	0.86
Critical	7.05	1.52	32.7	**0.013**
Cardiovascular and cerebrovascular diseases	5.03	1.88	13.43	**0.0013**

*Neutrophil count (10* ^*9*^ */L)*				
<40	1.33	0.26	6.87	0.73
40–75	1			
>75	4.21	1.37	12.94	**0.012**

*Aspartate aminotransferase, U/L*				
13–35	1			
>35	7.9	2.7	23.13	**0.0008**

Serum amyloid A (SAA), mg/L	1.01	1.006	1.02	**<0.0001**
Blood glucose, mmol/L	1.15	1.05	1.26	**0.0022**
*Diabetes treatment*				
Oral medication plus insulin	0.202	0.045	0.91	**0.037**
Oral medication only	0.063	0.014	0.28	**0.0003**
Insulin only	1			

*Antibiotics*				
Ganciclovir	5.27	1.2	23.21	**0.028**
Corticosteroid/glucocorticoid	10.31	2.32	45.76	**0.0022**

## Data Availability

The data can be provided after the article is published. The corresponding author (dengy@hawaii.edu) has the right to decide whether to share the data or not based on the research objectives and the plan provided.

## Supplementary Materials

Supplementary Materials Supplementary table S1: clinical characteristics of COVID-19 patients with and without T2D. Supplementary table S2: clinical characteristics between survivors and nonsurvivors in COVID-19 patients with T2D. Fig. S1: representative dynamic changes in chest computer tomography (CT) scans between admission and discharge for the three diabetes treatment groups. Fig. S2: survival analysis for the three diabetes treatment groups. Fig. S3: blood glucose levels of the three diabetes treatment groups.
