# Corrigendum to “Prognostic Factors for COVID-19 Hospitalized Patients with Preexisting Type 2 Diabetes”

**DOI:** 10.1155/2022/9838923

**Published:** 2022-07-04

**Authors:** Yuanyuan Fu, Ling Hu, Hong-Wei Ren, Yi Zuo, Shaoqiu Chen, Qiu-Shi Zhang, Chen Shao, Yao Ma, Lin Wu, Jun-Jie Hao, Chuan-Zhen Wang, Zhanwei Wang, Richard Yanagihara, Youping Deng

**Affiliations:** ^1^Department of Quantitative Health Sciences, John A. Burns School of Medicine, University of Hawaii at Manoa, Honolulu, HI, USA; ^2^Tianyou Hospital, Affiliated to Wuhan University of Science and Technology, Wuhan, Hubei, China; ^3^Cancer Epidemiology Program, University of Hawaii Cancer Center, University of Hawaii at Manoa, Honolulu, HI, USA; ^4^Department of Pediatrics, John A. Burns School of Medicine, University of Hawaii at Manoa, Honolulu, HI, USA

In the article titled “Prognostic Factors for COVID-19 Hospitalized Patients with Preexisting Type 2 Diabetes” [1], the authors would like to update the Authors' Contributions section to note the equal contribution of Yuanyuan Fu and Ling Hu. The updated section is as follows.
