# Supplementary material for: Prognostic Factors for COVID-19 Hospitalized Patients with Preexisting Type 2 Diabetes
Source: Int J Endocrinol. 2022 Jan 17;2022:9322332. doi: 10.1155/2022/9322332 (PMC8763039; doi:10.1155/2022/9322332)
Supplement: Supplementary Materials — Supplementary table S1: clinical characteristics of COVID-19 patients with and without T2D. Supplementary table S2: clinical characteristics between survivors and nonsurvivors in COVID-19 patients with T2D. Fig. S1: representative dynamic changes in chest computer tomography (CT) scans between admission and discharge for the three diabetes treatment groups. Fig. S2: survival analysis for the three diabetes treatment groups. Fig. S3: blood glucose levels of the three diabetes treatment groups. [file 9322332.f1.zip › 9322332.f1/11_Supplementary_Table_S1_20211213.docx]

**Supplementary Table S1. Clinical characteristics of COVID-19 patients with and without T2D**

| **Variables** | **Total**  **(N=382)** | **Non-diabetes (N=274)** | **Diabetes (N=108)** | ***P* value** |
| --- | --- | --- | --- | --- |
| **Characteristics** |  |  |  |  |
| **Age (Median, IQR)** | 63(52-70) | 60(48-67) | 68(61-75) | **<0.0001** |
| **Sex** |  |  |  | 0.72 |
| Female | 189(49.48) | 134(48.91) | 55(50.93) |  |
| Male | 193(50.52) | 140(51.09) | 53(49.07) |  |
| **Occupation** |  |  |  | **<0.0001** |
| Employed | 116(30.37) | 103(37.59) | 13(12.04) |  |
| Retired | 161(42.15) | 111(40.51) | 50(46.30) |  |
| Unemployed | 105(27.49) | 60(21.90) | 45(41.66) |  |
| **BMI** |  |  |  | 0.07 |
| <25 | 275(80.17) | 194(77.29) | 81(88.04) |  |
| 25-30 | 55(16.03) | 47(18.73) | 8(8.70) |  |
| ≥30 | 13(3.79) | 10(3.98) | 3(3.26) |  |
| **Smoking history** |  |  |  | 0.63 |
| No | 342(89.53) | 244(89.05) | 98(90.74) |  |
| Yes | 40(10.47) | 30(10.95) | 10(9.26) |  |
| **Onset time (Median, IQR)** | 9(6-13) | 9(6-12) | 10(5-15) | 0.06 |
| **Use of hypnotics** |  |  |  | **0.016** |
| No | 258(75.00) | 175(71.43) | 83(83.84) |  |
| Yes | 86(25.00) | 70(28.57) | 16(16.16) |  |
| **Signs and symptoms** |  |  |  |  |
| Fever | 306(80.10) | 234(85.40) | 72(66.67) | **<0.0001** |
| Cough | 202(52.88) | 135(49.27) | 67(62.04) | **0.024** |
| Fever and Cough | 265(69.37) | 207(75.55) | 58(53.70) | **<0.0001** |
| Chest distress | 8(2.09) | 2(0.73) | 6(5.56) | **0.0076** |
| Nausea and vomiting | 4(1.05) | 0 | 4(1.05) | **0.0061** |
| Dyspnea | 17(4.55) | 15(4.38) | 2(4.63) | 1 |
| **Coexisting conditions** |  |  |  |  |
| Any comorbidity | 138(36.31) | 69(25.18) | 69(63.89) | **<0.0001** |
| Cirrhosis | 4(1.05) | 3(1.09) | 1(0.93) | 1 |
| Hypertension | 144(37.70) | 79(28.83) | 65(60.19) | **<0.0001** |
| Cerebrovascular disease | 14(3.66) | 4(1.46) | 10(9.26) | **0.0008** |
| Cardiovascular and cerebrovascular diseases | 48(12.57) | 27(9.85) | 21(19.44) | **0.011** |
| Digestive system disease | 24(6.28) | 16(5.84) | 8(7.41) | **0.57** |
| Endocrine system disease | 30(7.85) | 7(2.55) | 23(21.30) | **<0.0001** |
| Respiratory system disease | 32(8.30) | 25(9.12) | 7(6.48) | 0.4 |
| **Disease severity** |  |  |  | **<0.0001** |
| Non-severe | 160(41.88) | 128(46.72) | 32(29.63) |  |
| Severe | 189(49.48) | 132(48.18) | 57(52.78) |  |
| Critical | 33(8.64) | 14(5.11) | 19(17.59) |  |
| **Outcome** |  |  |  | **0.033** |
| Alive | 345(90.31) | 253(92.34) | 92(85.19) |  |
| Dead | 37(9.69) | 21(7.66) | 16(14.81) |  |
| **Radiologic and laboratory findings** |  |  |  |  |
| **Abnormalities on chest CT** |  |  |  |  |
| No GGO | 22(5.76) | 7(2.55) | 15(13.89) | **<0.0001** |
| Local GGO | 22(5.76) | 12(4.38) | 10(9.26) | 0.065 |
| Bilateral GGO | 191(50.00) | 147(53.65) | 44(40.74) | **0.023** |
| Combination of patchy ground glass opacity and pulmonary consolidation | 69(18.06) | 54(19.71) | 15(13.89) | 0.18 |
| Crazy paving sign | 29(7.59) | 21(7.66) | 8(7.41) | 0.93 |
| Diffuse patchy ground glass and air bronchogram | 29(7.59) | 16(5.84) | 13(12.04) | 0.039 |
| Bilateral multiple pulmonary consolidation and intralobular interstitial thickening | 10(2.62) | 6(2.19) | 4(3.70) | 0.48 |
| **Laboratory findings** |  |  |  |  |
| **White blood cell count, 10^9^/L** |  |  |  | **0.011** |
| <4 | 98(26.92) | 74(28.79) | 24(22.43) |  |
| 4-10 | 240(65.93) | 164(63.81) | 76(71.03) |  |
| >10 | 26(7.14) | 19(7.39) | 7(6.54) |  |
| **Neutrophil count , 10^9^/L** |  |  |  | **<0.0001** |
| <40 | 127(34.89) | 60(23.35) | 67(62.62) |  |
| 40-75 | 137(37.64) | 118(45.91) | 19(17.76) |  |
| >75 | 100(27.47) | 79(30.74) | 21(19.63) |  |
| **Lymphocyte count, 10^9^/L** |  |  |  | **<0.0001** |
| <20 | 238(65.38) | 147(57.20) | 91(85.05) |  |
| 20-50 | 122(33.52 | 106(41.25) | 16(14.95) |  |
| >50 | 4(1.10) | 4(1.56) | 0 |  |
| **Monocyte count, 10^9^/L** |  |  |  | **<0.0001** |
| <3 | 160(44.20) | 86(33.73) | 74(69.16) |  |
| 3-10 | 160(44.20) | 134(52.55) | 26(24.30) |  |
| >10 | 42(11.60) | 35(13.73) | 7(6.54) |  |
| **Platelet count, 10^9^/L** |  |  |  | 0.82 |
| <100 | 17(4.70) | 11(4.31) | 6(5.61) |  |
| 100-300 | 283(78.18) | 195(48.04) | 84(78.50) |  |
| >300 | 62(17.13) | 45(17.65) | 17(15.89) |  |
| **Alanine aminotransferase, U/L** |  |  |  | 0.11 |
| ≤7 | 44(11.83) | 36(13.48) | 8(7.62) |  |
| 7-40 | 266(71.51) | 183(68.54) | 83(79.05) |  |
| >40 | 62(16.67) | 48(17.98) | 14(13.33) |  |
| **Aspartate aminotransferase, U/L** |  |  |  | **0.029** |
| <13 | 50(13.40) | 41(15.30) | 9(8.57) |  |
| 13-35 | 227(60.86) | 152(56.72) | 75(71.43) |  |
| >35 | 96(25.74) | 75(27.99) | 21(20.00) |  |
| **C-reactive protein level, mg/L** | 26.7(8.0-62.6) | 28.8(11.1-65.9) | 19.2(2.8-59.5) | 0.37 |
| **Serum amyloid A (SAA) , mg/L** | 92.5(22.0-210.0) | 115(34-210) | 34(4-200) | **0.0016** |
| **Prothrombin time, s** | 11.9(11.1-13.0) | 11.8(11.0-12.9) | 12.0(11.3-13.4) | 0.36 |
| **Activated partial thromboplastin time, s** | 29.85(25.00-39.40) | 31.0(24.6-40.2) | 27.7(25.3-27.7) | 0.29 |
| **D-dimer, mg/L** | 0.38(0.01-1.73) | 0.29(0.01-1.80) | 0.50(0.09-1.65) | 0.13 |
| **Hypersensitive troponin I, pg/mL** | 0.008(0-0.041) | 0.008(0-0.059) | 0.007(0.001-0.0225) | **0.011** |
| **Creatine kinase–CMB, U/L** | 39.0(4.05-72.5) | 6.11(3.9-59.0) | 49.0(30.0-82.0) | 0.91 |
| **Lactate dehydrogenase, U/L** | 172.5(2.7-236.5) | 43.0(2.3-274.0) | 191.0(138.0-234.0) | 0.39 |
| **Total bilirubin, mmol/L** | 10(7-15) | 10(7-15) | 11(8-16) | 0.38 |
| **Blood urea nitrogen, mmol/L** | 3.5(2.7-4.8) | 4.6(3.3-6.9) | 5.5(4.2-7.9) | 0.16 |
| **Creatinine, μmol/L** | 63(48-79) | 60(46-76) | 68.5(52.0-87.0) | **0.0023** |
| **Procalcitonin, ng/mL** | 0.25(0.01-0.06) | 0.03(0.01-0.07) | 0.02(0.01-0.06) | **<0.0001** |
| **Blood glucose, mmol/L** | 5.8(5.1-7.5) | 5.5(4.9-6.4) | 7.7(5.9-10.8) | **<0.0001** |
| **Glycated hemoglobin, mmol/mol** |  |  |  |  |
| **Potassium, mmol/L** | 4.0(3.6-4.4) | 3.9(3.5-4.3) | 4.1(3.7-4.5) | **0.0073** |
| **Low-density lipoprotein (LDL), mmol/L** | 2.31(1.88-2.96 | 2.26(1.82-2.86) | 2.50(2.12-3.11) | **0.047** |
| **High-density lipoprotein (HDL), mmol/L** | 1.07(0.86-1.37 | 1.09(0.86-1.33) | 1.04(0.88-1.30) | 0.35 |
| **Treatment** |  |  |  |  |
| **Antiviral therapy** |  |  |  |  |
| Oseltamivir | 244(64.04) | 193(70.70) | 51(47.22) | **<0.0001** |
| Ganciclovir | 251(65.88) | 191(69.96) | 60(55.56) | **0.0075** |
| Arbidol | 250(65.62) | 180(65.93) | 80(64.81） | 0.83 |
| Kaletra | 30(7.87) | 18(6.59) | 12(11.11) | 0.14 |
| Interferon | 22(5.76) | 18(6.57) | 4(3.70) | 0.28 |
| **Antibiotic therapy** |  |  |  |  |
| Antibiotics | 344(90.29) | 259(94.87) | 85(78.70) | **<0.0001** |
| **Steroid therapy** |  |  |  |  |
| Corticosteroid/Glucocorticoid | 196(60.49) | 161(58.97) | 35(68.63) | 0.2 |
| **Oxygen support** |  |  |  |  |
| **Non-invasive ventilation** |  |  |  | **<0.0001** |
| No | 330(86.39) | 251(91.61) | 79(73.15) |  |
| Yes | 52(13.61) | 23(8.39) | 29(26.85) |  |
| **Invasive ventilation** |  |  |  | 0.52 |
| No | 264(69.11) | 192(70.07) | 72(66.67) |  |
| Yes | 118(30.89) | 82(29.93) | 36(33.33) |  |
| **Complications** |  |  |  |  |
| Acute cardiac injury | 44(11.52) | 30(10.95) | 14(12.96) | 0.58 |
| Arrhythmia | 28(7.35) | 13(4.76) | 15(13.89) | **0.0021** |
| ARDS* | 105(27.49) | 75(27.37) | 30(27.78) | 0.94 |
| Acute kidney injury | 13(3.41) | 8(2.93) | 5(4.63) | 0.53 |
| Acute renal injury | 17(4.46) | 11(4.03) | 6(5.56) | 0.52 |
| Septic shock | 19(4.99) | 8(2.93) | 11(10.19) | **0.0034** |
| Secondary infection | 10(2.62) | 5(1.83) | 5(4.63) | 0.15 |

*ARDS, Acute respiratory distress syndrome
