# Supplementary material for: Prognostic Factors for COVID-19 Hospitalized Patients with Preexisting Type 2 Diabetes
Source: Int J Endocrinol. 2022 Jan 17;2022:9322332. doi: 10.1155/2022/9322332 (PMC8763039; doi:10.1155/2022/9322332)
Supplement: Supplementary Materials — Supplementary table S1: clinical characteristics of COVID-19 patients with and without T2D. Supplementary table S2: clinical characteristics between survivors and nonsurvivors in COVID-19 patients with T2D. Fig. S1: representative dynamic changes in chest computer tomography (CT) scans between admission and discharge for the three diabetes treatment groups. Fig. S2: survival analysis for the three diabetes treatment groups. Fig. S3: blood glucose levels of the three diabetes treatment groups. [file 9322332.f1.zip › 9322332.f1/12_Supplementary_Table S2_20211213.docx]

**Supplementary Table S2. Clinical characteristics between survivors and non-survivors in COVID-19 patients with T2D**

| **Variables** | **Total**  **(N=108)** | **Non-survivor (N=16)** | **Survivor (N=92)** | ***P* Value** |
| --- | --- | --- | --- | --- |
| **Characteristics** |  |  |  |  |
| **Age, y** | 68(61-75) | 74(65-89) | 67(60-73) | 0.017 |
| **Age at diagnosed with diabetes, y** | 60(52-66) | 62(56-67) | 60(51-66) | 0.14 |
| **Duration of diabetes, y** | 8(4-13) | 10(6-20) | 8(4-12) | 0.051 |
| **Sex** |  |  |  | 0.53 |
| Female | 55(50.9) | 7(43.8) | 48(52.2) |  |
| Male | 53(49.1) | 9(56.2) | 44(47.8) |  |
| **Occupation** |  |  |  | 0.17 |
| Employed | 13(12.0) | 1(6.3) | 12(13.0) |  |
| Retired | 50(46.3) | 11(68.7) | 39(42.4) |  |
| Unemployed | 45(41.7) | 4(25.0) | 41(44.6) |  |
| **BMI** |  |  |  | 0.053 |
| <25 | 81(88.0) | 12(85.7) | 69(88.5) |  |
| 25-30 | 8(8.7) | 0 | 8(10.2) |  |
| ≥30 | 3(3.3) | 2(14.3) | 1(1.3) |  |
| **Smoking history** |  |  |  | 0.16 |
| No | 98(90.7) | 13(81.3) | 85(92.4) |  |
| Yes | 10(9.3) | 3(18.7) | 7(7.6) |  |
| **Onset time, d** | 10(5-15) | 8(4-11) | 10(6-15) | 0.032 |
| **Disease severity** |  |  |  | 0.0003 |
| Non-severe | 32(29.6) | 2(12.5) | 30(32.6) |  |
| Severe | 57(52.8) | 5(31.3) | 52(56.5) |  |
| Critical | 19(17.6) | 9(56.2) | 10(10.9) |  |
| **Signs and symptoms** |  |  |  |  |
| Fever | 72/108(66.7) | 9/16(56.3) | 63/92(68.5) | 0.34 |
| Cough | 67/108(62.0) | 9/16(56.3) | 58/92(63.0) | 0.59 |
| Fever and Cough | 50/108(53.7) | 7/16(43.7) | 51/92(55.4) | 0.43 |
| Chest distress | 6/108(5.6) | 2/16(12.5) | 4/92(4.4) | 0.21 |
| Nausea and vomiting | 4/108(3.7) | 1/16(6.3) | 3/92(85.2) | 0.48 |
| Dyspnea | 5/108(4.6) | 0/16(0) | 5/92(5.4) |  |
| **Coexisting conditions** |  |  |  |  |
| Any comorbidity | 69/108(63.9) | 14/16(87.5) | 55/92(59.8) | 0.033 |
| Cirrhosis | 1/108(0.9) | 1/16(6.3) | 0/92(0) | 0.29 |
| Hypertension | 65/108(60.2) | 10/16(62.5) | 55/92(59.8) | 1 |
| Cerebrovascular disease | 10/108(9.3) | 0/16(0) | 10/92(10.9) | 0.35 |
| Cardiovascular and cerebrovascular diseases | 21/108(19.4) | 8/16(50.0) | 13/92(14.1) | 0.0027 |
| Digestive system disease | 8/108(7.4) | 4/16(25.0) | 4/92(4.4) | 0.016 |
| Endocrine system disease | 23/108(21.3) | 1/16(6.3) | 22/92(23.9) | 0.18 |
| Respiratory system disease | 7/108(6.5) | 3/16(18.8) | 4/92(4.4) | 0.065 |
|  |  |  |  |  |
| **Radiologic and laboratory findings** |  |  |  |  |
| **Radiologic findings** |  |  |  |  |
| **Abnormalities on chest CT** |  |  |  |  |
| No GGO | 15/108(13.9) | 1/16(6.3) | 14/92(15.2) | 0.46 |
| Local GGO | 10/108(9.3) | 0/16(0) | 10/92(10.9) | 0.35 |
| Bilateral GGO | 44/108(40.7) | 6/16(37.5) | 38/92(41.3) | 1 |
| Combination of patchy ground glass opacity and pulmonary consolidation | 15/108(13.9) | 0/16(0) | 15/92(16.3) | 0.12 |
| Crazy waving sign | 8/108(7.4) | 3/16(18.8) | 5/92(5.4) | 0.095 |
| Diffuse patchy ground glass and air bronchogram | 13/108(12.0) | 1/16(6.3) | 12/92(13.0) | 0.69 |
| Bilateral multiple pulmonary consolidation and intralobular interstitial thickening | 4/108(3.7) | 2/16(12.5) | 2/92(2.7) | 0.1 |
| **Laboratory findings** |  |  |  |  |
| **White blood cell count, 10^9^/L** |  |  |  | 0.011 |
| <4 | 24(22.4) | 3(20.0) | 21(22.8) |  |
| 4-10 | 76(71.0) | 8(53.3) | 68(73.9) |  |
| >10 | 7(6.5) | 4(26.7) | 3(3.3) |  |
| **Neutrophil count, 10^9^/L** |  |  |  | 0.0029 |
| <40 | 67(62.6) | 5(33.3) | 62(67.4) |  |
| 40-75 | 19(17.8) | 2(13.3) | 17(18.5) |  |
| >75 | 21(19.6) | 8(53.4) | 13(14.1) |  |
| **Lymphocyte count, 10^9^/L** |  |  |  | 0.46 |
| <20 | 91(85.1) | 14(93.3) | 77(83.7) |  |
| 20-50 | 16(14.9) | 1(6.7) | 15(16.3) |  |
| **Monocyte count, 10^9^/L** |  |  |  | 0.39 |
| <3 | 74(69.2) | 9(60.0) | 65(70.7) |  |
| 3-10 | 26(24.3) | 4(26.7) | 22(23.9) |  |
| >10 | 7(6.5) | 2(13.3) | 5(5.4) |  |
| **Platelet count, 10^9^/L** |  |  |  | 0.036 |
| <100 | 6(5.6) | 3(20.0) | 3(3.3) |  |
| 100-300 | 84(78.5) | 9(60.0) | 75(81.5) |  |
| >300 | 17(15.9) | 3(20.0) | 14(15.2) |  |
| **Alanine aminotransferase, U/L** |  |  |  | 0.21 |
| ≤7 | 8(7.6) | 0 | 8(8.9) |  |
| 7-40 | 83(79.1) | 11(73.3) | 72(80.0) |  |
| >40 | 14(13.3) | 4(26.7) | 10(11.1) |  |
| **Aspartate aminotransferase, U/L** |  |  |  | <0.0001 |
| <13 | 9(8.6) | 0 | 9(10.0) |  |
| 13-35 | 75(71.4) | 5(33.3) | 70(77.8) |  |
| >35 | 21(20.0) | 10(66.7) | 11(12.2) |  |
| **C-reactive protein, mg/L** | 19.2(2.8-59.5) | 94.85(48-146.75) | 15.2(2.4-45) | <0.0001 |
| **Serum amyloid A (SAA), mg/L** | 34(4-200) | 210(200-250) | 153(2-153.5) | **<**0.0001 |
| **Prothrombin time, s** | 12(11.3-13.4) | 12.05(11.1-12.8) | 12(10.7-13.4) | 0.2 |
| **Activated partial thromboplastic time, s** | 27.7(25.3-34.2) | 35.35(18.20-57) | 26.9(25.1-30.8) | 0.0044 |
| **D-dimer, mg/L** | 0.5(0.09-1.65) | 0.33(0.01-3.54) | 0.5(0.13-1.56) | 0.011 |
| **Hypersensitive troponin I, pg/mL** | 0.007(0.001-0.023) | 0.039(0.01-0.13) | 0.001(0-0.017) | 0.18 |
| **Creatine kinase–CMB, U/L** | 49(30-82) | 40(12.7-122.5) | 51(34.0-81.5) | 0.24 |
| **Lactate dehydrogenase, U/L** | 191(138-234) | 232(223-525) | 176(136.5-228.5) | 0.0011 |
| **Total bilirubin, mmol/L** | 11(8-16) | 12(8-18) | 11(8-14) | 0.62 |
| **Blood urea nitrogen, mmol/L** | 5.45(4.15-7.85) | 7.9(6.1-10.2) | 5.1(4.0-6.7) | 0.18 |
| **Creatinine, μmol/L** | 68.5(52-87) | 74(66-116) | 68(49-86) | 0.026 |
| **Procalcitonin, ng/mL** | 0.02(0.01-0.06) | 0.05(0.02-0.23) | 0.02(0.01-0.05) | 0.35 |
| **Blood glucose, mmol/L** | 7.7(5.9-10.8) | 11.5(5.4-18.3) | 7.6(6.1-9.5) | 0.05 |
| **Glycated hemoglobin, mmol/mol** | 7.5(6.5-8.6) | 7.7(6.6-8.6) | 7.5(6.5-8.6) | 0.73 |
| **Potassium, mmol/L** | 4.1(3.7-4.5) | 4.2(3.4-4.6) | 4.1(3.7-4.5) | 0.47 |
| **Low-density lipoprotein (LDL), mmol/L** | 2.5(2.1-3.1) | 2.3(2.1-2.8) | 2.6(2.1-3.2) | 0.012 |
| **High-density lipoprotein (HDL), mmol/L** | 1.04(0.9-1.3) | 1.05(0.8-1.2) | 1.03(0.9-1.3) | 0.013 |
|  |  |  |  |  |
| **Treatments** |  |  |  |  |
| **Diabetes treatment** |  |  |  | <0·0001 |
| Oral medication plus insulin | 15(13.9) | 2(12.5) | 13(14.1) |  |
| Oral medication only | 65(60.2) | 2(12.5) | 63(68.5) |  |
| Insulin only | 28(25.9) | 12(75.0) | 16(17.4) |  |
| **Antiviral therapy** |  |  |  |  |
| Oseltamivir | 51/108(47.2) | 12/16(75.0) | 39/92(85.2) | 0.028 |
| Ganciclovir | 60/108(55.6) | 14/16(87.5) | 46/92(50.0) | 0.0059 |
| Arbidol | 70/108(64.8) | 7/16(43.8) | 63/92(68.5) | 0.056 |
| Kaletra | 12/108(11.1) | 4/16(25.0) | 8/92(8.7) | 0.077 |
| Interferon | 4/108(3.7) | 2/16(12.5) | 2/92(2.2) | 0.1 |
| **Antibiotic therapy** |  |  |  |  |
| Antibiotics | 85/108(78.7) | 16/16(100) | 69/92(75.0) | 0.021 |
| **Steroid therapy** |  |  |  |  |
| Corticosteroid/Glucocorticoid | 35/95(36.9) | 13/15(86.7) | 22/80(27.5) | <0.0001 |
| **Oxygen support** |  |  |  |  |
| **Non-invasive ventilation** |  |  |  | <0.0001 |
| No | 79(73.2) | 4(25.0) | 75(81.5) |  |
| Yes | 29(26.8) | 12(75.0) | 17(18.5) |  |
| **Invasive ventilation** |  |  |  | <0.0001 |
| No | 72(66.7) | 2(12.5) | 70(76.1) |  |
| Yes | 36(33.3) | 14(87.5) | 22(23.9) |  |
|  |  |  |  |  |
| **Complication** |  |  |  |  |
| Any complication | 33(30.5) | 17(18.5) | 16(100) | <0.0001 |
| Acute cardiac injury | 14/108(13.0) | 10/16(62.5) | 4/92(4.4) | <0.0001 |
| Arrhythmia | 15/108(13.9) | 8/16(50.0) | 7/92(7.6) | 0.0001 |
| ARDS^*^ | 30/108(27.8) | 15/16(93.6) | 15/92(16.3) | <0.0001 |
| Acute kidney injury | 5/108(4.6) | 2/16(12.5) | 3/92(3.3) | 0.16 |
| Septic shock | 11/108(10.2) | 8/16(50.0) | 3/92(3.3) | <0.0001 |
| Secondary infection | 5/108(4.6) | 5/16(31.3) | 0/92(00 | <0.0001 |
| * Acute respiratory distress syndrome |  |  |  |  |
