# Supplementary material for: Prognostic Factors for COVID-19 Hospitalized Patients with Preexisting Type 2 Diabetes
Source: Int J Endocrinol. 2022 Jan 17;2022:9322332. doi: 10.1155/2022/9322332 (PMC8763039; doi:10.1155/2022/9322332)
Supplement: Supplementary Materials — Supplementary table S1: clinical characteristics of COVID-19 patients with and without T2D. Supplementary table S2: clinical characteristics between survivors and nonsurvivors in COVID-19 patients with T2D. Fig. S1: representative dynamic changes in chest computer tomography (CT) scans between admission and discharge for the three diabetes treatment groups. Fig. S2: survival analysis for the three diabetes treatment groups. Fig. S3: blood glucose levels of the three diabetes treatment groups. [file 9322332.f1.zip › 9322332.f1/13_supplementary legends_Fig S123_20211213.docx]

**Supplementary Figure Legends**

**Fig. S1 Representative dynamic changes in chest computer tomography (CT) scans between admission and discharge for the three diabetes treatment groups**

(A1-A2) CT images of a 65-year-old male patient, whose blood glucose at admission was 9.4 mmol/L, and treated with metformin during hospitalization. A1 (day 1): lungs showed patchy ground glass shadows, and the internal blood vessels were running naturally; A2 (day 12): lung lesions were less and the boundary was clearer than before. (B1-B2) CT images of a 60-year-old male patient, whose blood glucose at admission was 8 mmol/L, and treated with acarbose and insulin during hospitalization. B1 (day1): lungs showed scattered patchy, cord shape fuzzy shadow, some parts showing ground glass density; B2 (day 12): density of lung lesions were decreased. (C1-C2) CT images of a 55-year-old male patient, whose blood glucose at admission was 18.1 mmol/L, treated with insulin during hospitalization, and died. C1 (day1): both lungs showed scattered patchy ground glass shadows, some lesions were consolidated, and air bronchi signs were seen inside; C2 (day 4): foci of the two lung diseases were more advanced, and most of the lesions were solid, with air bronchi signs inside and a small amount of effusion in the left thoracic cavity.

**Fig. S2 Survival analysis for three diabetes treatment groups**

(A) Kaplan-Meier curves demonstrating survival of COVID-19 patients with T2D by diabetes treatment. *P* values for survival analysis were derived by the log-rank test.

**Fig. S3 Blood glucose level on three diabetes treatment groups**

(A) Distribution of blood glucose in three diabetes treatment groups was significantly different (Mean±SD, *p*=0.0025).
