# Supplementary material for: Prognostic Factors for COVID-19 Hospitalized Patients with Preexisting Type 2 Diabetes
Source: Int J Endocrinol. 2022 Jan 17;2022:9322332. doi: 10.1155/2022/9322332 (PMC8763039; doi:10.1155/2022/9322332)
Supplement: Supplementary Materials — Supplementary table S1: clinical characteristics of COVID-19 patients with and without T2D. Supplementary table S2: clinical characteristics between survivors and nonsurvivors in COVID-19 patients with T2D. Fig. S1: representative dynamic changes in chest computer tomography (CT) scans between admission and discharge for the three diabetes treatment groups. Fig. S2: survival analysis for the three diabetes treatment groups. Fig. S3: blood glucose levels of the three diabetes treatment groups. [file 9322332.f1.zip › 9322332.f1/Supplementary_material.pdf]

Supplementary Table S1. Clinical characteristics of COVID-19 patients with and without T2D

Variables	Total (N=382)	Non-diabetes (N=274)	Diabetes (N=108)	*P* value
**Characteristics**				
**Age (Median, IQR)**	63(52-70)	60(48-67)	68(61-75)	**<0.0001**
**Sex**				0.72
Female	189(49.48)	134(48.91)	55(50.93)	
Male	193(50.52)	140(51.09)	53(49.07)	
**Occupation**				**<0.0001**
Employed	116(30.37)	103(37.59)	13(12.04)	
Retired	161(42.15)	111(40.51)	50(46.30)	
Unemployed	105(27.49)	60(21.90)	45(41.66)	
**BMI**				0.07
<25	275(80.17)	194(77.29)	81(88.04)	
25-30	55(16.03)	47(18.73)	8(8.70)	
≥30	13(3.79)	10(3.98)	3(3.26)	
**Smoking history**				0.63
No	342(89.53)	244(89.05)	98(90.74)	
Yes	40(10.47)	30(10.95)	10(9.26)	
**Onset time (Median, IQR)**	9(6-13)	9(6-12)	10(5-15)	0.06
**Use of hypnotics**				**0.016**
No	258(75.00)	175(71.43)	83(83.84)	
Yes	86(25.00)	70(28.57)	16(16.16)	
**Signs and symptoms**				
Fever	306(80.10)	234(85.40)	72(66.67)	**<0.0001**
Cough	202(52.88)	135(49.27)	67(62.04)	**0.024**
Fever and Cough	265(69.37)	207(75.55)	58(53.70)	**<0.0001**
Chest distress	8(2.09)	2(0.73)	6(5.56)	**0.0076**
Nausea and vomiting	4(1.05)	0	4(1.05)	**0.0061**
Dyspnea	17(4.55)	15(4.38)	2(4.63)	1
**Coexisting conditions**				
Any comorbidity	138(36.31)	69(25.18)	69(63.89)	**<0.0001**
Cirrhosis	4(1.05)	3(1.09)	1(0.93)	1
Hypertension	144(37.70)	79(28.83)	65(60.19)	**<0.0001**
Cerebrovascular disease	14(3.66)	4(1.46)	10(9.26)	**0.0008**
Cardiovascular and cerebrovascular diseases	48(12.57)	27(9.85)	21(19.44)	**0.011**
Digestive system disease	24(6.28)	16(5.84)	8(7.41)	**0.57**
Endocrine system disease	30(7.85)	7(2.55)	23(21.30)	**<0.0001**
Respiratory system disease	32(8.30)	25(9.12)	7(6.48)	0.4
**Disease severity**				**<0.0001**
Non-severe	160(41.88)	128(46.72)	32(29.63)	
Severe	189(49.48)	132(48.18)	57(52.78)	
Critical	33(8.64)	14(5.11)	19(17.59)	
**Outcome**				**0.033**

Alive	345(90.31)	253(92.34)	92(85.19)	
Dead	37(9.69)	21(7.66)	16(14.81)	
**Radiologic and laboratory findings**				
**Abnormalities on chest CT**				
No GGO	22(5.76)	7(2.55)	15(13.89)	**<0.0001**
Local GGO	22(5.76)	12(4.38)	10(9.26)	0.065
Bilateral GGO	191(50.00)	147(53.65)	44(40.74)	**0.023**
Combination of patchy ground glass opacity and pulmonary consolidation	69(18.06)	54(19.71)	15(13.89)	0.18
Crazy paving sign	29(7.59)	21(7.66)	8(7.41)	0.93
Diffuse patchy ground glass and air bronchogram	29(7.59)	16(5.84)	13(12.04)	0.039
Bilateral multiple pulmonary consolidation and intralobular interstitial thickening	10(2.62)	6(2.19)	4(3.70)	0.48
**Laboratory findings**				
**White blood cell count, 10^9/L**				**0.011**
<4	98(26.92)	74(28.79)	24(22.43)	
4-10	240(65.93)	164(63.81)	76(71.03)	
>10	26(7.14)	19(7.39)	7(6.54)	
**Neutrophil count , 10^9/L**				**<0.0001**
<40	127(34.89)	60(23.35)	67(62.62)	
40-75	137(37.64)	118(45.91)	19(17.76)	
>75	100(27.47)	79(30.74)	21(19.63)	
**Lymphocyte count, 10^9/L**				**<0.0001**
<20	238(65.38)	147(57.20)	91(85.05)	
20-50	122(33.52	106(41.25)	16(14.95)	
>50	4(1.10)	4(1.56)	0	
**Monocyte count, 10^9/L**				**<0.0001**
<3	160(44.20)	86(33.73)	74(69.16)	
3-10	160(44.20)	134(52.55)	26(24.30)	
>10	42(11.60)	35(13.73)	7(6.54)	
**Platelet count, 10^9/L**				0.82
<100	17(4.70)	11(4.31)	6(5.61)	
100-300	283(78.18)	195(48.04)	84(78.50)	
>300	62(17.13)	45(17.65)	17(15.89)	
**Alanine aminotransferase, U/L**				0.11
≤7	44(11.83)	36(13.48)	8(7.62)	
7-40	266(71.51)	183(68.54)	83(79.05)	
>40	62(16.67)	48(17.98)	14(13.33)	
**Aspartate aminotransferase, U/L**				**0.029**
<13	50(13.40)	41(15.30)	9(8.57)	
13-35	227(60.86)	152(56.72)	75(71.43)	
>35	96(25.74)	75(27.99)	21(20.00)	
**C-reactive protein level, mg/L**	26.7(8.0-62.6)	28.8(11.1-65.9)	19.2(2.8-59.5)	0.37
**Serum amyloid A (SAA) , mg/L**	92.5(22.0-210.0)	115(34-210)	34(4-200)	**0.0016**
**Prothrombin time, s**	11.9(11.1-13.0)	11.8(11.0-12.9)	12.0(11.3-13.4)	0.36
**Activated partial thromboplastin time, s**	29.85(25.00-39.40)	31.0(24.6-40.2)	27.7(25.3-27.7)	0.29
**D-dimer, mg/L**	0.38(0.01-1.73)	0.29(0.01-1.80)	0.50(0.09-1.65)	0.13

**Hypersensitive troponin I, pg/mL**	0.008(0-0.041)	0.008(0-0.059)	0.007(0.001-0.0225)	**0.011**
**Creatine kinase–CMB, U/L**	39.0(4.05-72.5)	6.11(3.9-59.0)	49.0(30.0-82.0)	0.91
**Lactate dehydrogenase, U/L**	172.5(2.7-236.5)	43.0(2.3-274.0)	191.0(138.0-234.0)	0.39
**Total bilirubin, mmol/L**	10(7-15)	10(7-15)	11(8-16)	0.38
**Blood urea nitrogen, mmol/L**	3.5(2.7-4.8)	4.6(3.3-6.9)	5.5(4.2-7.9)	0.16
**Creatinine, μmol/L**	63(48-79)	60(46-76)	68.5(52.0-87.0)	**0.0023**
**Procalcitonin, ng/mL**	0.25(0.01-0.06)	0.03(0.01-0.07)	0.02(0.01-0.06)	**<0.0001**
**Blood glucose, mmol/L**	5.8(5.1-7.5)	5.5(4.9-6.4)	7.7(5.9-10.8)	**<0.0001**
**Glycated hemoglobin, mmol/mol**				
**Potassium, mmol/L**	4.0(3.6-4.4)	3.9(3.5-4.3)	4.1(3.7-4.5)	**0.0073**
**Low-density lipoprotein (LDL), mmol/L**	2.31(1.88-2.96	2.26(1.82-2.86)	2.50(2.12-3.11)	**0.047**
**High-density lipoprotein (HDL), mmol/L**	1.07(0.86-1.37	1.09(0.86-1.33)	1.04(0.88-1.30)	0.35
**Treatment**				
**Antiviral therapy**				
Oseltamivir	244(64.04)	193(70.70)	51(47.22)	**<0.0001**
Ganciclovir	251(65.88)	191(69.96)	60(55.56)	**0.0075**
Arbidol	250(65.62)	180(65.93)	80(64.81)	0.83
Kaletra	30(7.87)	18(6.59)	12(11.11)	0.14
Interferon	22(5.76)	18(6.57)	4(3.70)	0.28
**Antibiotic therapy**				
Antibiotics	344(90.29)	259(94.87)	85(78.70)	**<0.0001**
**Steroid therapy**				
Corticosteroid/Glucocorticoid	196(60.49)	161(58.97)	35(68.63)	0.2
**Oxygen support**				
**Non-invasive ventilation**				**<0.0001**
No	330(86.39)	251(91.61)	79(73.15)	
Yes	52(13.61)	23(8.39)	29(26.85)	
**Invasive ventilation**				0.52
No	264(69.11)	192(70.07)	72(66.67)	
Yes	118(30.89)	82(29.93)	36(33.33)	
**Complications**				
Acute cardiac injury	44(11.52)	30(10.95)	14(12.96)	0.58
Arrhythmia	28(7.35)	13(4.76)	15(13.89)	**0.0021**
ARDS*	105(27.49)	75(27.37)	30(27.78)	0.94
Acute kidney injury	13(3.41)	8(2.93)	5(4.63)	0.53
Acute renal injury	17(4.46)	11(4.03)	6(5.56)	0.52
Septic shock	19(4.99)	8(2.93)	11(10.19)	**0.0034**
Secondary infection	10(2.62)	5(1.83)	5(4.63)	0.15

*ARDS, Acute respiratory distress syndrome

Supplementary Table S2. Clinical characteristics between survivors and non-survivors in COVID-19 patients with T2D

Variables	Total (N=108)	Non-survivor (N=16)	Survivor (N=92)	*P* Value
**Characteristics**				
**Age, y**	68(61-75)	74(65-89)	67(60-73)	0.017
**Age at diagnosed with diabetes, y**	60(52-66)	62(56-67)	60(51-66)	0.14
**Duration of diabetes, y**	8(4-13)	10(6-20)	8(4-12)	0.051
**Sex**				0.53
Female	55(50.9)	7(43.8)	48(52.2)	
Male	53(49.1)	9(56.2)	44(47.8)	
**Occupation**				0.17
Employed	13(12.0)	1(6.3)	12(13.0)	
Retired	50(46.3)	11(68.7)	39(42.4)	
Unemployed	45(41.7)	4(25.0)	41(44.6)	
**BMI**				0.053
<25	81(88.0)	12(85.7)	69(88.5)	
25-30	8(8.7)	0	8(10.2)	
≥30	3(3.3)	2(14.3)	1(1.3)	
**Smoking history**				0.16
No	98(90.7)	13(81.3)	85(92.4)	
Yes	10(9.3)	3(18.7)	7(7.6)	
**Onset time, d**	10(5-15)	8(4-11)	10(6-15)	0.032
**Disease severity**				0.0003
Non-severe	32(29.6)	2(12.5)	30(32.6)	
Severe	57(52.8)	5(31.3)	52(56.5)	
Critical	19(17.6)	9(56.2)	10(10.9)	
**Signs and symptoms**				
Fever	72/108(66.7)	9/16(56.3)	63/92(68.5)	0.34
Cough	67/108(62.0)	9/16(56.3)	58/92(63.0)	0.59
Fever and Cough	50/108(53.7)	7/16(43.7)	51/92(55.4)	0.43
Chest distress	6/108(5.6)	2/16(12.5)	4/92(4.4)	0.21
Nausea and vomiting	4/108(3.7)	1/16(6.3)	3/92(85.2)	0.48
Dyspnea	5/108(4.6)	0/16(0)	5/92(5.4)	
**Coexisting conditions**				
Any comorbidity	69/108(63.9)	14/16(87.5)	55/92(59.8)	0.033
Cirrhosis	1/108(0.9)	1/16(6.3)	0/92(0)	0.29
Hypertension	65/108(60.2)	10/16(62.5)	55/92(59.8)	1
Cerebrovascular disease	10/108(9.3)	0/16(0)	10/92(10.9)	0.35
Cardiovascular and cerebrovascular diseases	21/108(19.4)	8/16(50.0)	13/92(14.1)	0.0027
Digestive system disease	8/108(7.4)	4/16(25.0)	4/92(4.4)	0.016
Endocrine system disease	23/108(21.3)	1/16(6.3)	22/92(23.9)	0.18
Respiratory system disease	7/108(6.5)	3/16(18.8)	4/92(4.4)	0.065
**Radiologic and laboratory findings**				
**Radiologic findings**				
**Abnormalities on chest CT**				

No GGO	15/108(13.9)	1/16(6.3)	14/92(15.2)	0.46
Local GGO	10/108(9.3)	0/16(0)	10/92(10.9)	0.35
Bilateral GGO	44/108(40.7)	6/16(37.5)	38/92(41.3)	1
Combination of patchy ground glass opacity and pulmonary consolidation	15/108(13.9)	0/16(0)	15/92(16.3)	0.12
Crazy waving sign	8/108(7.4)	3/16(18.8)	5/92(5.4)	0.095
Diffuse patchy ground glass and air bronchogram	13/108(12.0)	1/16(6.3)	12/92(13.0)	0.69
Bilateral multiple pulmonary consolidation and intralobular interstitial thickening	4/108(3.7)	2/16(12.5)	2/92(2.7)	0.1
**Laboratory findings**				
**White blood cell count, 10^9/L**				0.011
<4	24(22.4)	3(20.0)	21(22.8)	
4-10	76(71.0)	8(53.3)	68(73.9)	
>10	7(6.5)	4(26.7)	3(3.3)	
**Neutrophil count, 10^9/L**				0.0029
<40	67(62.6)	5(33.3)	62(67.4)	
40-75	19(17.8)	2(13.3)	17(18.5)	
>75	21(19.6)	8(53.4)	13(14.1)	
**Lymphocyte count, 10^9/L**				0.46
<20	91(85.1)	14(93.3)	77(83.7)	
20-50	16(14.9)	1(6.7)	15(16.3)	
**Monocyte count, 10^9/L**				0.39
<3	74(69.2)	9(60.0)	65(70.7)	
3-10	26(24.3)	4(26.7)	22(23.9)	
>10	7(6.5)	2(13.3)	5(5.4)	
**Platelet count, 10^9/L**				0.036
<100	6(5.6)	3(20.0)	3(3.3)	
100-300	84(78.5)	9(60.0)	75(81.5)	
>300	17(15.9)	3(20.0)	14(15.2)	
**Alanine aminotransferase, U/L**				0.21
≤7	8(7.6)	0	8(8.9)	
7-40	83(79.1)	11(73.3)	72(80.0)	
>40	14(13.3)	4(26.7)	10(11.1)	
**Aspartate aminotransferase, U/L**				<0.0001
<13	9(8.6)	0	9(10.0)	
13-35	75(71.4)	5(33.3)	70(77.8)	
>35	21(20.0)	10(66.7)	11(12.2)	
**C-reactive protein, mg/L**	19.2(2.8-59.5)	94.85(48-146.75)	15.2(2.4-45)	<0.0001
**Serum amyloid A (SAA), mg/L**	34(4-200)	210(200-250)	153(2-153.5)	**<**0.0001
**Prothrombin time, s**	12(11.3-13.4)	12.05(11.1-12.8)	12(10.7-13.4)	0.2
**Activated partial thromboplastic time, s**	27.7(25.3-34.2)	35.35(18.20-57)	26.9(25.1-30.8)	0.0044
**D-dimer, mg/L**	0.5(0.09-1.65)	0.33(0.01-3.54)	0.5(0.13-1.56)	0.011
**Hypersensitive troponin I, pg/mL**	0.007(0.001-0.023)	0.039(0.01-0.13)	0.001(0-0.017)	0.18
**Creatine kinase–CMB, U/L**	49(30-82)	40(12.7-122.5)	51(34.0-81.5)	0.24
**Lactate dehydrogenase, U/L**	191(138-234)	232(223-525)	176(136.5-228.5)	0.0011
**Total bilirubin, mmol/L**	11(8-16)	12(8-18)	11(8-14)	0.62
**Blood urea nitrogen, mmol/L**	5.45(4.15-7.85)	7.9(6.1-10.2)	5.1(4.0-6.7)	0.18

**Creatinine, μmol/L**	68.5(52-87)	74(66-116)	68(49-86)	0.026
**Procalcitonin, ng/mL**	0.02(0.01-0.06)	0.05(0.02-0.23)	0.02(0.01-0.05)	0.35
**Blood glucose, mmol/L**	7.7(5.9-10.8)	11.5(5.4-18.3)	7.6(6.1-9.5)	0.05
**Glycated hemoglobin, mmol/mol**	7.5(6.5-8.6)	7.7(6.6-8.6)	7.5(6.5-8.6)	0.73
**Potassium, mmol/L**	4.1(3.7-4.5)	4.2(3.4-4.6)	4.1(3.7-4.5)	0.47
**Low-density lipoprotein (LDL), mmol/L**	2.5(2.1-3.1)	2.3(2.1-2.8)	2.6(2.1-3.2)	0.012
**High-density lipoprotein (HDL), mmol/L**	1.04(0.9-1.3)	1.05(0.8-1.2)	1.03(0.9-1.3)	0.013
**Treatments**				
**Diabetes treatment**				<0·0001
Oral medication plus insulin	15(13.9)	2(12.5)	13(14.1)	
Oral medication only	65(60.2)	2(12.5)	63(68.5)	
Insulin only	28(25.9)	12(75.0)	16(17.4)	
**Antiviral therapy**				
Oseltamivir	51/108(47.2)	12/16(75.0)	39/92(85.2)	0.028
Ganciclovir	60/108(55.6)	14/16(87.5)	46/92(50.0)	0.0059
Arbidol	70/108(64.8)	7/16(43.8)	63/92(68.5)	0.056
Kaletra	12/108(11.1)	4/16(25.0)	8/92(8.7)	0.077
Interferon	4/108(3.7)	2/16(12.5)	2/92(2.2)	0.1
**Antibiotic therapy**				
Antibiotics	85/108(78.7)	16/16(100)	69/92(75.0)	0.021
**Steroid therapy**				
Corticosteroid/Glucocorticoid	35/95(36.9)	13/15(86.7)	22/80(27.5)	<0.0001
**Oxygen support**				
**Non-invasive ventilation**				<0.0001
No	79(73.2)	4(25.0)	75(81.5)	
Yes	29(26.8)	12(75.0)	17(18.5)	
**Invasive ventilation**				<0.0001
No	72(66.7)	2(12.5)	70(76.1)	
Yes	36(33.3)	14(87.5)	22(23.9)	
**Complication**				
Any complication	33(30.5)	17(18.5)	16(100)	<0.0001
Acute cardiac injury	14/108(13.0)	10/16(62.5)	4/92(4.4)	<0.0001
Arrhythmia	15/108(13.9)	8/16(50.0)	7/92(7.6)	0.0001
ARDS*	30/108(27.8)	15/16(93.6)	15/92(16.3)	<0.0001
Acute kidney injury	5/108(4.6)	2/16(12.5)	3/92(3.3)	0.16
Septic shock	11/108(10.2)	8/16(50.0)	3/92(3.3)	<0.0001
Secondary infection	5/108(4.6)	5/16(31.3)	0/92(00	<0.0001

* Acute respiratory distress syndrome

Fig. S1

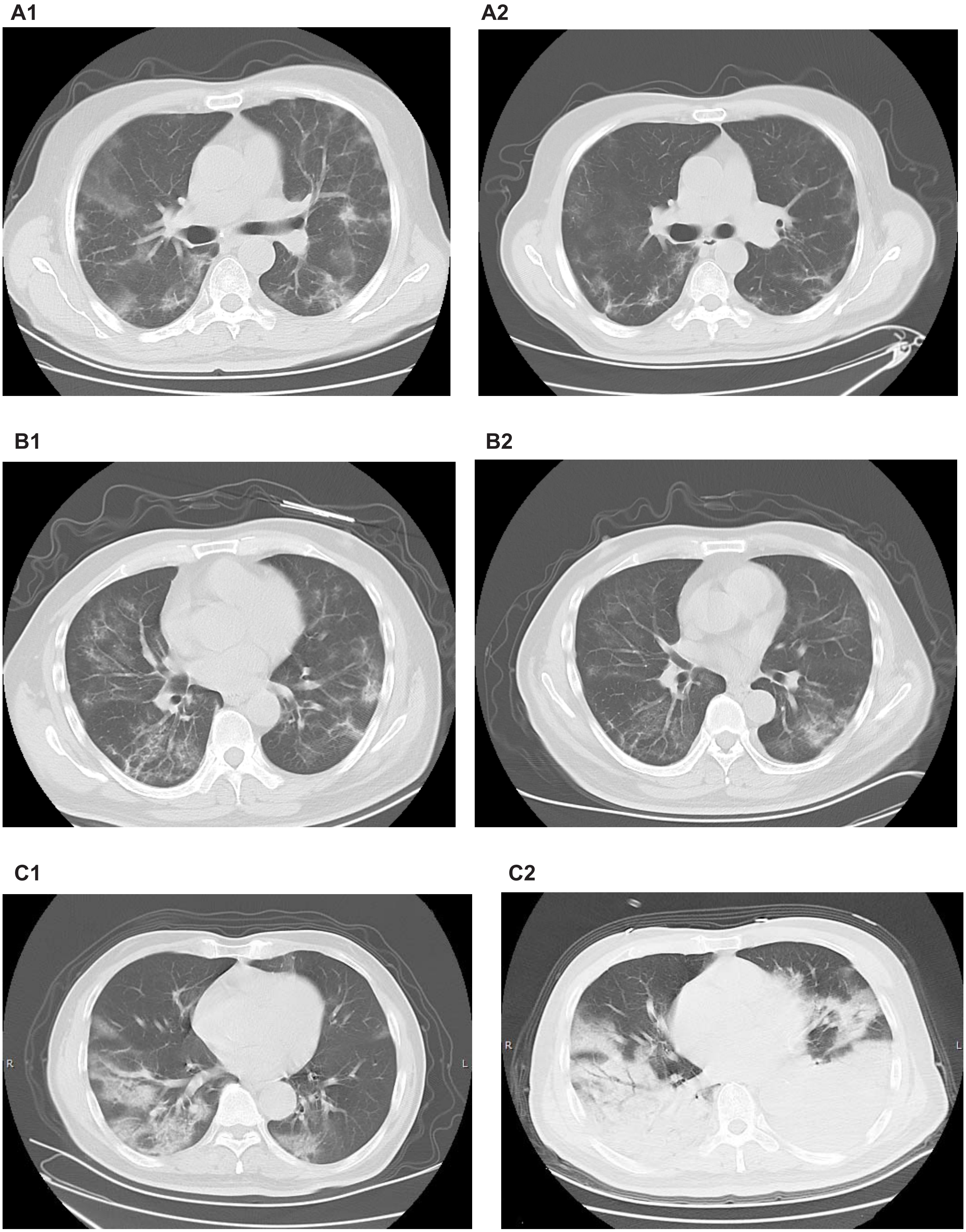

Fig. S2

A

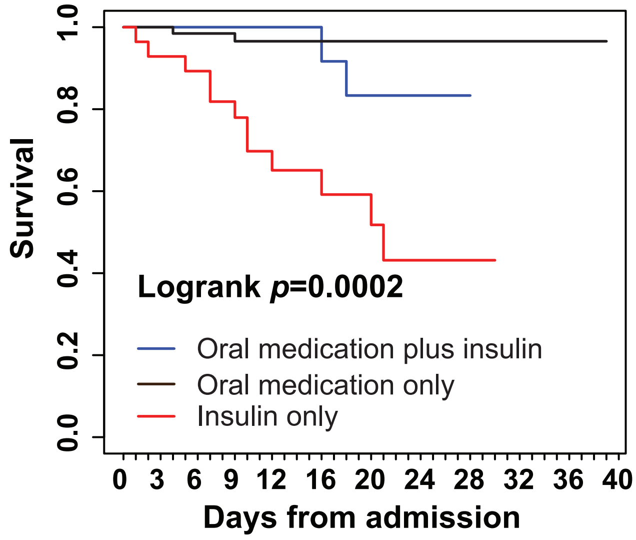
Survival
1.0
0.8
0.6
0.4
0.2
0.0
Logrank p=0.0002
Oral medication plus insulin
Oral medication only
Insulin only
0 3 6 9 12 16 20 24 28 32 36 40
Days from admission

Fig. S3

A

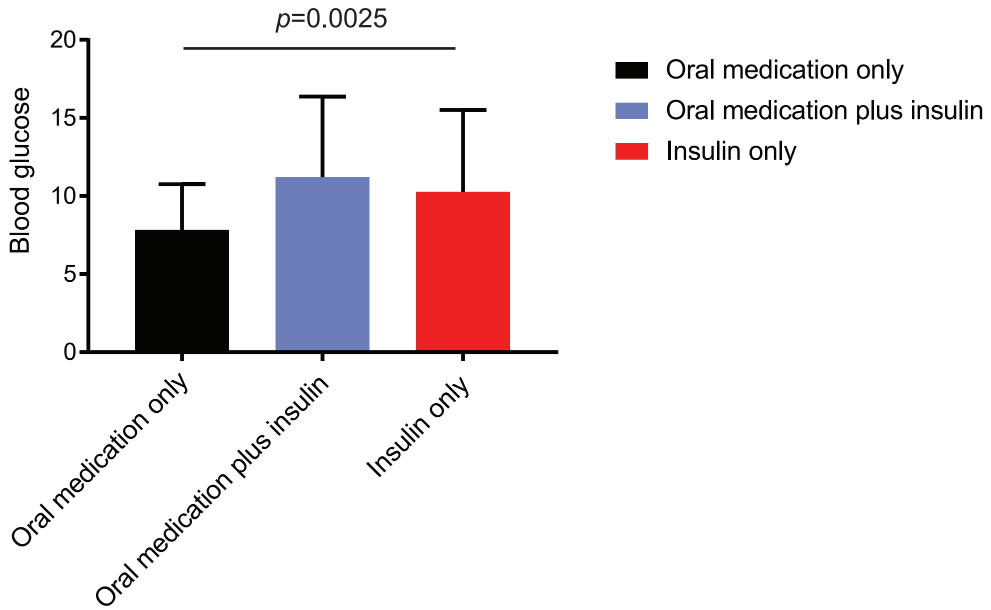

p=0.0025
Blood glucose
20
15
10
5
0
Oral medication only
Oral medication plus insulin
Insulin only
Oral medication only
Oral medication plus insulin
Insulin only

Supplementary Figure Legends

Fig. S1 Representative dynamic changes in chest computer tomography (CT) scans between admission and discharge for the three diabetes treatment groups

(A1-A2) CT images of a 65-year-old male patient, whose blood glucose at admission was 9.4 mmol/L, and treated with metformin during hospitalization. A1 (day 1): lungs showed patchy ground glass shadows, and the internal blood vessels were running naturally; A2 (day 12): lung lesions were less and the boundary was clearer than before. (B1-B2) CT images of a 60-year-old male patient, whose blood glucose at admission was 8 mmol/L, and treated with acarbose and insulin during hospitalization. B1 (day1): lungs showed scattered patchy, cord shape fuzzy shadow, some parts showing ground glass density; B2 (day 12): density of lung lesions were decreased. (C1-C2) CT images of a 55-year-old male patient, whose blood glucose at admission was 18.1 mmol/L, treated with insulin during hospitalization, and died. C1 (day1): both lungs showed scattered patchy ground glass shadows, some lesions were consolidated, and air bronchi signs were seen inside; C2 (day 4): foci of the two lung diseases were more advanced, and most of the lesions were solid, with air bronchi signs inside and a small amount of effusion in the left thoracic cavity.

Fig. S2 Survival analysis for three diabetes treatment groups

(A) Kaplan-Meier curves demonstrating survival of COVID-19 patients with T2D by diabetes treatment. *P* values for survival analysis were derived by the log-rank test.

Fig. S3 Blood glucose level on three diabetes treatment groups

(A) Distribution of blood glucose in three diabetes treatment groups was significantly different (Mean±SD, p=0.0025).
