# Supplementary figures and images for: Prognostic Factors for COVID-19 Hospitalized Patients with Preexisting Type 2 Diabetes
Source: Int J Endocrinol. 2022 Jan 17;2022:9322332. doi: 10.1155/2022/9322332 (PMC8763039; doi:10.1155/2022/9322332)

Fig. S3

A

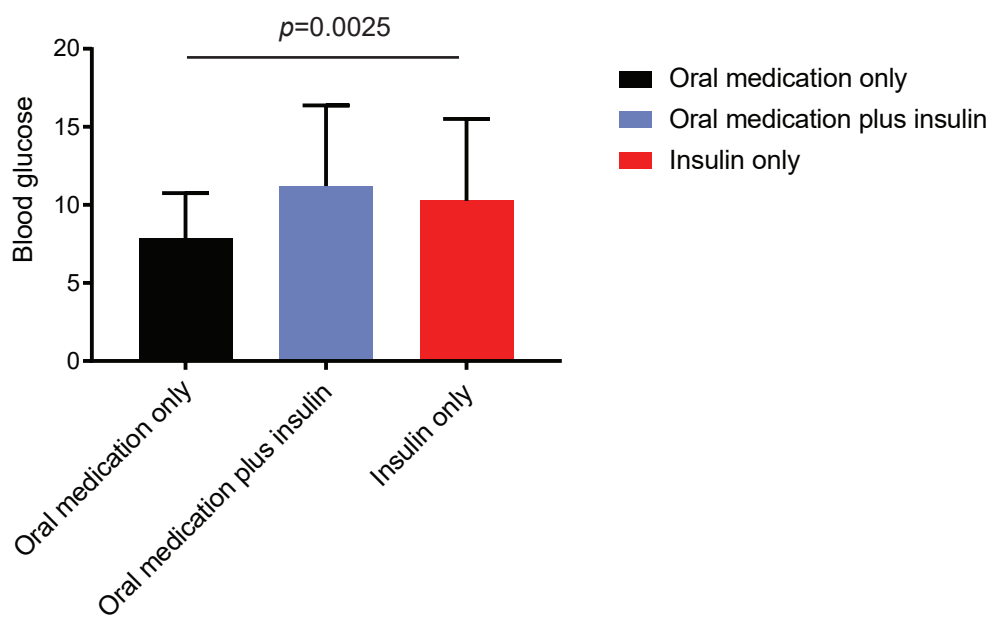

Supplement: Supplementary Materials — Supplementary table S1: clinical characteristics of COVID-19 patients with and without T2D. Supplementary table S2: clinical characteristics between survivors and nonsurvivors in COVID-19 patients with T2D. Fig. S1: representative dynamic changes in chest computer tomography (CT) scans between admission and discharge for the three diabetes treatment groups. Fig. S2: survival analysis for the three diabetes treatment groups. Fig. S3: blood glucose levels of the three diabetes treatment groups. [file 9322332.f1.zip › 9322332.f1/10_Supplementary_Figure_S3_20211213.pdf]

**Fig. S1**

**A1**

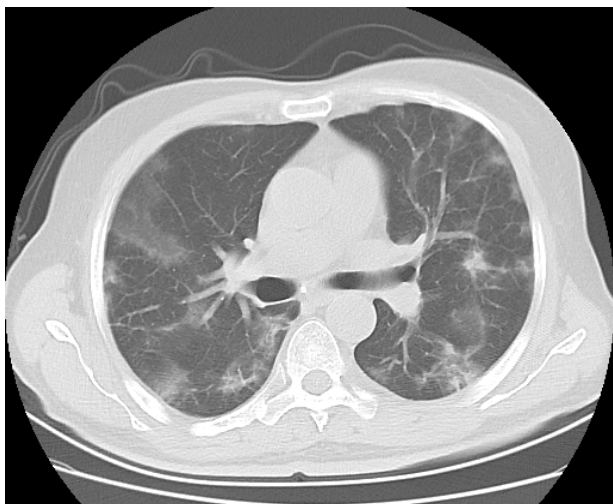

**A2**

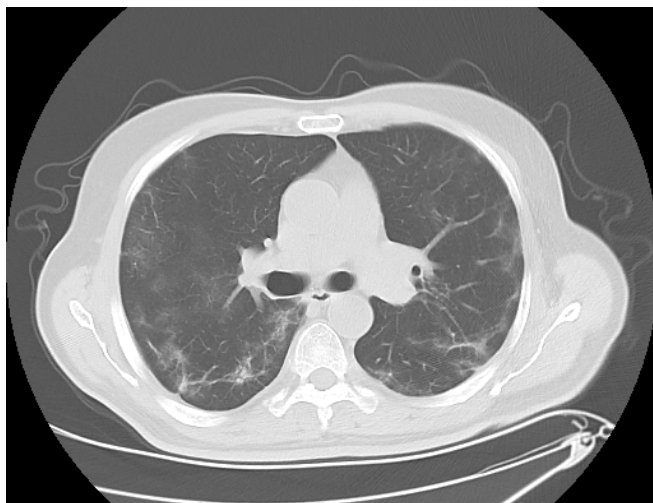

**B1**

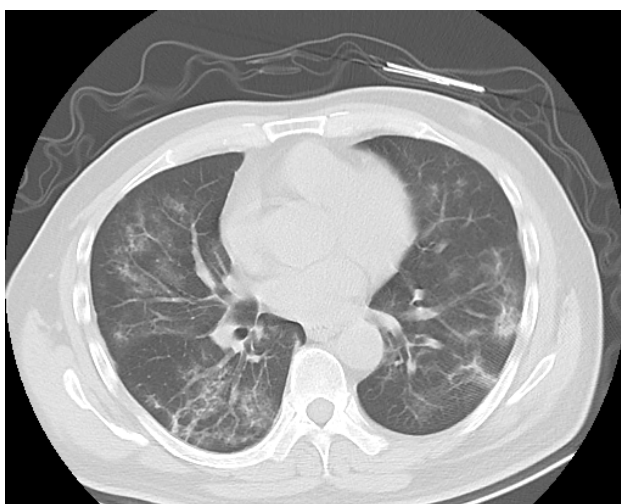

**B2**

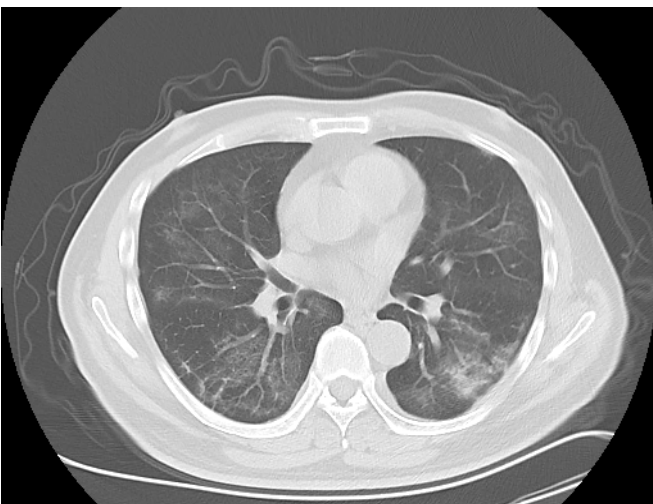

**C1**

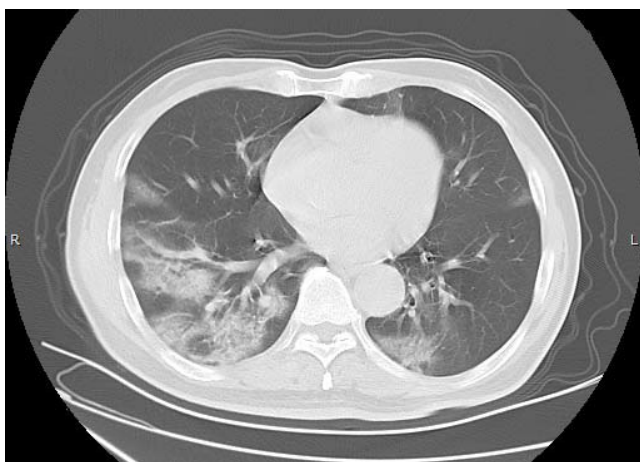

**C2**

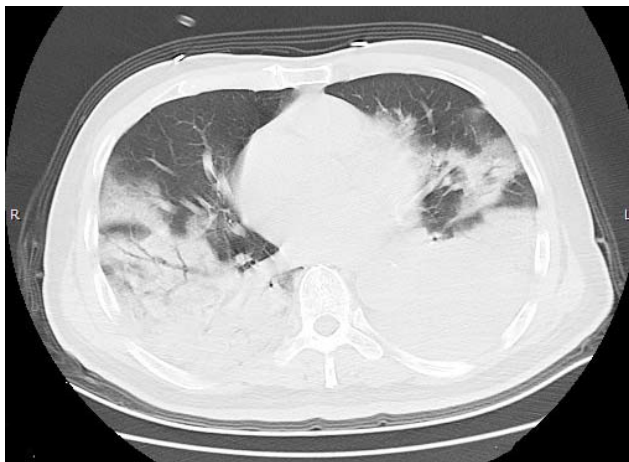

Supplement: Supplementary Materials — Supplementary table S1: clinical characteristics of COVID-19 patients with and without T2D. Supplementary table S2: clinical characteristics between survivors and nonsurvivors in COVID-19 patients with T2D. Fig. S1: representative dynamic changes in chest computer tomography (CT) scans between admission and discharge for the three diabetes treatment groups. Fig. S2: survival analysis for the three diabetes treatment groups. Fig. S3: blood glucose levels of the three diabetes treatment groups. [file 9322332.f1.zip › 9322332.f1/8_Supplementary_Figure_S1_20211213.pdf]

Fig. S2

A

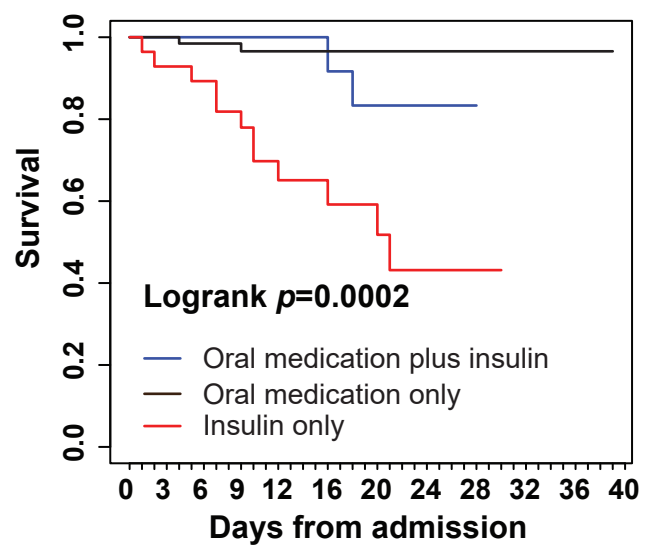

Supplement: Supplementary Materials — Supplementary table S1: clinical characteristics of COVID-19 patients with and without T2D. Supplementary table S2: clinical characteristics between survivors and nonsurvivors in COVID-19 patients with T2D. Fig. S1: representative dynamic changes in chest computer tomography (CT) scans between admission and discharge for the three diabetes treatment groups. Fig. S2: survival analysis for the three diabetes treatment groups. Fig. S3: blood glucose levels of the three diabetes treatment groups. [file 9322332.f1.zip › 9322332.f1/9_Supplementary_Figure_S2_20211213.pdf]
